# The Mars 2020 *Perseverance* Rover Mast Camera Zoom (Mastcam-Z) Multispectral, Stereoscopic Imaging Investigation

**DOI:** 10.1007/s11214-020-00755-x

**Published:** 2021-02-15

**Authors:** J. F. Bell, J. N. Maki, G. L. Mehall, M. A. Ravine, M. A. Caplinger, Z. J. Bailey, S. Brylow, J. A. Schaffner, K. M. Kinch, M. B. Madsen, A. Winhold, A. G. Hayes, P. Corlies, C. Tate, M. Barrington, E. Cisneros, E. Jensen, K. Paris, K. Crawford, C. Rojas, L. Mehall, J. Joseph, J. B. Proton, N. Cluff, R. G. Deen, B. Betts, E. Cloutis, A. J. Coates, A. Colaprete, K. S. Edgett, B. L. Ehlmann, S. Fagents, J. P. Grotzinger, C. Hardgrove, K. E. Herkenhoff, B. Horgan, R. Jaumann, J. R. Johnson, M. Lemmon, G. Paar, M. Caballo-Perucha, S. Gupta, C. Traxler, F. Preusker, M. S. Rice, M. S. Robinson, N. Schmitz, R. Sullivan, M. J. Wolff

**Affiliations:** 1 Arizona State Univ., Tempe, AZ USA; 2 JPL/Caltech, Pasadena, CA USA; 3 Malin Space Science Systems, Inc., San Diego, CA USA; 4 Univ. of Copenhagen, Copenhagen, Denmark; 5 Cornell Univ., Ithaca, NY USA; 6 Opscode LLC, Healdsburg, CA USA; 7 The Planetary Society, Pasadena, CA USA; 8 Univ. of Winnipeg, Winnipeg, Canada; 9 Mullard Space Science Laboratory, Univ. College, London, UK; 10 NASA/Ames Research Center, Moffett Field, CA USA; 11 Caltech, Pasadena, CA USA; 12 Univ. of Hawaii, Honolulu, HI USA; 13 USGS Astrogeology Science Center, Flagstaff, AZ USA; 14 Purdue Univ., South Bend, IN USA; 15 Inst. of Geological Sciences, Free University Berlin, Berlin, Germany; 16 APL/Johns Hopkins Univ., Laurel, MD USA; 17 Space Science Inst., Boulder, CO USA; 18 Joanneum Research, Graz, Austria; 19 Imperial College, London, UK; 20 VRVis Research Center, Vienna, Austria; 21 DLR/German Aerospace Center, Berlin, Germany; 22 Western Washington Univ., Bellingham, WA USA

**Keywords:** Mars, Mars 2020 mission, *Perseverance* rover, Jezero crater, Space instrumentation, Space imaging

## Abstract

Mastcam-Z is a multispectral, stereoscopic imaging investigation on the Mars 2020 mission’s *Perseverance* rover. Mastcam-Z consists of a pair of focusable, 4:1 zoomable cameras that provide broadband red/green/blue and narrowband 400-1000 nm color imaging with fields of view from 25.6° × 19.2° (26 mm focal length at 283 μrad/pixel) to 6.2° × 4.6° (110 mm focal length at 67.4 μrad/pixel). The cameras can resolve (≥ 5 pixels) ∼0.7 mm features at 2 m and ∼3.3 cm features at 100 m distance. Mastcam-Z shares significant heritage with the Mastcam instruments on the Mars Science Laboratory *Curiosity* rover. Each Mastcam-Z camera consists of zoom, focus, and filter wheel mechanisms and a 1648 × 1214 pixel charge-coupled device detector and electronics. The two Mastcam-Z cameras are mounted with a 24.4 cm stereo baseline and 2.3° total toe-in on a camera plate ∼2 m above the surface on the rover’s Remote Sensing Mast, which provides azimuth and elevation actuation. A separate digital electronics assembly inside the rover provides power, data processing and storage, and the interface to the rover computer. Primary and secondary Mastcam-Z calibration targets mounted on the rover top deck enable tactical reflectance calibration. Mastcam-Z multispectral, stereo, and panoramic images will be used to provide detailed morphology, topography, and geologic context along the rover’s traverse; constrain mineralogic, photometric, and physical properties of surface materials; monitor and characterize atmospheric and astronomical phenomena; and document the rover’s sample extraction and caching locations. Mastcam-Z images will also provide key engineering information to support sample selection and other rover driving and tool/instrument operations decisions.

## Introduction

Mars was a habitable world. That profound and stunning finding is a product of the past ∼25 years of strategic exploration using robotic orbiters, landers, and rovers that have revolutionized our understanding of the Red Planet. A series of fantastically successful missions have acquired the key global, regional, and local-scale data sets necessary to map the planet’s geology, chemistry, and mineralogy, and to interpret those results in the context of Martian geologic and environmental history (*e.g.*, Malin and Edgett 2001; Squyres et al. 2004; Grotzinger et al. 2014; Ehlmann and Edwards 2014). Most of these new missions, experiments, and investigations were guided by the results of their predecessors, and some of these missions continue to operate to this day.

The scientific discoveries from the Mars Exploration Rovers (MER) *Spirit* and *Opportunity*, and from the Mars Science Laboratory (MSL) rover *Curiosity*, in particular, tell a story of a planet hypothesized to have had a more Earthlike (though never the same as Earth) early history. The details and timing of the transition from the early Martian Noachian climate through the transitional Hesperian and into the modern, cold and arid Amazonian are the subject of intense debate within the science community. Significantly, MER, MSL, and orbiter science results from several missions indicate that sub-surface diagenetic and hydrothermal fluids, near-surface groundwater, and even surface liquid water altered primarily basaltic precursor crustal materials, leaving behind tell-tale evidence of the alteration environment’s properties, such as pH and water-to-rock ratio, in the form of diverse, widespread hydrated minerals like sulfates, carbonates, and phyllosilicates (see, *e.g.*, reviews in Bell 2008; Ehlmann and Edwards 2014; Bishop et al. 2020).

The Mars 2020 mission and its rover, *Perseverance*, will build on these discoveries and be the critical next step in NASA’s strategic Mars exploration program (Farley et al. 2020). Collection of data essential to addressing the mission’s scientific goals (Table 1) requires observations by a visible color, multispectral, and stereo imaging system with the capability to conduct lateral and stratigraphic surveys and analyses at multiple spatial scales on many targets, as well as to assist in rover navigation. Imager system mission roles include characterizing the geological context along the rover traverse to help select locations for further in-depth analyses and sampling by arm-mounted instruments and documenting and validating the success of those arm-related activities (Mustard et al. 2013).

**Table 1:** The Mastcam-Z Investigation and Mars 2020 Mission Objectives

Mars 2020 mission objectives^a^		Mastcam-Z goals^b^	Rationale
A. Characterize the processes that formed and modified the geologic record within a field exploration area on Mars selected for evidence of an astrobiologically-relevant ancient environment and geologic diversity		1. Geomorphology, Processes, Geologic Record	Identifying geomorphic features and their constituent igneous and sedimentary structures at both coarse and fine scales helps to reconstruct the local and regional paleoenvironment, as well as reveal the role that water played in its evolution.
		2. Current Atmospheric and Astronomical Conditions and Events	Documenting surface/atmospheric interactions, including aeolian processes and meteorologic events (*e.g.*, dust storms and clouds) provides essential context for interpreting geologic structures and assessing habitability.
		3. Operational Support	Characterizing potential target materials and their geologic context provides essential complementary data to other instrument investigations as well as tactical operations.
B. Perform the following astrobiologically relevant investigations on the geologic materials at the landing site:	1. Determine the habitability of an ancient environment 2. For ancient environments interpreted to have been habitable, search for materials with high biosignature preservation potential 3. Search for potential evidence of past life using the observations regarding habitability and preservation as a guide	1. Geomorphology, Processes, Geologic Record	Identifying textures and compositional changes in rocks and outcrops from previously water-rich environments provides essential context for determining the history of deposition, diagenesis, and erosion required to determine habitability and biosignature preservation potential.
		3. Operational Support	Characterizing target materials and their surrounding terrain provides essential context to complementary instrument investigations. Remote identification of targets facilitates tactical operation of analytical and contact instruments.
C. Assemble a returnable cache of samples for possible future return to Earth	1. Obtain samples that are scientifically selected, for which the field context is documented, that contain the most promising samples identified in Objective B and that represent the geologic diversity of the field site 2. Ensure compliance with future needs in the areas of planetary protection and engineering so that the cache could be returned in the future if NASA chooses to do so	1. Geomorphology, Processes, Geologic Record	Documenting the geologic features found in the vicinity of cached samples is essential to the interpretation of measurements made by analytic and/or contact instruments, as well as potential laboratory studies following sample retrieval.
		2. Current Atmospheric and Astronomical Conditions and Events	Atmospheric monitoring (*e.g.*, aeolian activity, clouds, aerosols) provides engineering constraints for potential cache retrieval and document the conditions the cache apparatus will be subjected to prior to recovery.
		3. Operational Support	Characterizing target materials and their surrounding terrain provides essential context to complementary instrument investigations. Documenting the three-dimensional structure of the cache area is essential to the design of a potential recovery mission.
D. Contribute to the preparation for human exploration of Mars by making significant progress towards filling at least one major Strategic Knowledge Gap	1. Demonstration of In-Situ Resource Utilization (ISRU) technologies to enable propellant and consumable oxygen production from the Martian atmosphere for future exploration 2. Characterization of atmospheric dust size and morphology to understands its effects on the operation of surface systems and human health 3. Surface weather measurements to validate global atmospheric models	1. Geomorphology, Processes, Geologic Record	Observing the properties and dominant transport processes affecting local fines, frost, and ice provides environmental constraints for surface systems associated with human exploration.
		2. Current Atmospheric and Astronomical Conditions and Events	Tracking atmospheric and meteorological events / processes (*e.g.*, dust events, clouds, aerosols, etc.) both provides ground-truth for general circulation models and documents environmental conditions relevant to human exploration.
		3. Operational Support	Monitoring and characterizing Helicopter and MOXIE experiments or demonstrations helps document instrument health and provide temporal context for the interpretation of results.

^a^See Farley et al. 2020.

^b^See Sect. 2.2 for the full descriptions of Mastcam-Z investigation goals.

The *Perseverance* rover’s Mast Camera Zoom (Mastcam-Z) instrument and science/operations investigation is designed to provide these essential imaging observations to help meet the Mars 2020 mission’s goals. Mastcam-Z is a high-heritage imaging system based directly on the successful MSL Mastcam investigation (Malin et al. 2017) with all of the capabilities of the MSL Mastcam instruments but augmented by a 4:1 zoom capability that will significantly enhance its stereoscopic imaging performance for science, rover navigation, and *in situ* instrument and tool placement support. The Mastcam-Z camera heads are a matched pair of zoomable, focusable, charge-coupled device (CCD) cameras that can each collect broad-band red/green/blue (RGB) or narrow-band visible/near-infrared (VNIR) color data as well as direct solar images using neutral density filters. Each camera has a selectable field of view ranging from ∼7.7° to ∼31.9° diagonally, with the ability to resolve features ∼0.7 mm in size in the near field and ∼3.3 cm in size at 100 m distance (assuming that “resolve” requires those features to be ≥5 pixels in size) from its position ∼2 m above the surface on the *Perseverance* Remote Sensing Mast (RSM).

Mastcam-Z will observe textural, mineralogical, structural, and morphologic details in rocks and fines at the rover’s field site in Jezero crater (Stack et al. 2020). Imaging from Mastcam-Z and many of the 23 other cameras on the rover and its systems (Maki et al. 2020) will permit the science team to constrain rock type (e.g., sedimentary vs. igneous) and texture, and to assemble a geologic history of the site from stratigraphic clues in outcrops and regolith. The Mastcam-Z cameras will also document dynamic processes and events via video (*e.g.*, aeolian sand movement, dust devils, cloud motions, and astronomical phenomena) at video rates of 4 frames/sec or faster for subframes, observe the Sun and sky for atmospheric science, and contribute imaging and video data to rover navigation and target selection for investigations by the rover’s mobility, coring, and sample caching subsystems as well as other instruments.

Because of Mastcam-Z’s MSL heritage, no new technologies were developed for this enhanced *Perseverance* hardware implementation. Rather, we leveraged high-heritage parts, designs, and accommodation solutions from MSL, as well as science and operational lessons-learned from that mission and others, to maximize the overall science return for Mars 2020 (including detailed documentation and context of the potentially-returnable samples being cached) with only modest modifications to the specific implementation of existing MSL-flown technologies. The capability to zoom (which is new), focus, acquire data at high-speed, perform limited onboard data processing within the system’s own flight software, and store large amounts of data in the system’s own internal buffers provides numerous options to maximize operational efficiency. These capabilities also permit investigators to examine targets in detail that are otherwise out of the rover’s reach, and to view near-field rocks, sedimentary and igneous structures, and fines (regolith, aeolian deposits) at a pixel scale as small as ∼133 μm/pixel at a distance of 2 m.

Here we describe the Mastcam-Z imaging system, the flight hardware of which consists of two camera heads mounted on the rover’s altitude/azimuth actuated RSM, one Digital Electronics Assembly (DEA) within the rover body, and two small grayscale and color calibration targets mounted on the rover’s deck. We also describe the specific scientific investigation that will directly support Mars 2020 mission objectives and rover engineering operations, and our plans to acquire, process, and calibrate Mastcam-Z images and archive all image data in the NASA Planetary Data System (PDS). Companion papers provide substantial, additional details on the performance and pre-flight calibration of the Mastcam-Z cameras (Hayes et al. 2021) and on the design, pre-flight characterization, and intended uses of the Mastcam-Z Primary and Secondary calibration targets (Kinch et al. 2020a).

## Mastcam-Z Investigation Goals

### High-Level Goals

Mastcam-Z investigation goals (Table 1) and associated observational objectives (Table 2) respond directly to and will support the achievement of Objectives A-D of the NASA Mars 2020 mission (Farley et al. 2020). Achieving the Mastcam-Z goals and objectives will also enable the collection and analysis of data sets that can provide substantial ground truth for and/or synergy with current and planned Mars orbiter science investigations of the surface and atmosphere, near-term planned landed investigations like that of the European Space Agency’s 2022 ExoMars rover (Vago et al. 2017) and others, and future orbiters and landers/rovers that would be engaged in the planned Mars Sample Return campaign designed to bring back to Earth the samples collected and cached by the Mars 2020 mission (*e.g.*, Grady 2020).

**Table 2:** Mastcam-Z detailed goals and objectives

Mastcam-Z goals	Mastcam-Z detailed investigation objectives
1. Characterize the overall landscape geomorphology, processes, and the nature of the geologic record (mineralogy, texture, structure, stratigraphy) at the rover field site	1-a. Characterize the morphology, texture, and multispectral properties of rocks and outcrops to assess emplacement history, variability of composition, and physical properties.
	1-b. Determine the structure and orientation of stratigraphic boundaries, layers, and other key morphologic features to investigate emplacement and modification history.
	1-c. Characterize the position, size, morphology, texture, and multispectral properties of rocks and fines to constrain provenance and weathering history.
	1-d. Observe and monitor terrains disturbed by rover wheels and other hardware elements to assess surface to physical and chemical weathering.
	1-e. Distinguish among bedform types within the vicinity of the rover to evaluate the modification history of the landscape.
	1-f. Identify diagnostic sedimentary structures to determine emplacement history.
	1-g. Characterize finer scale color/spectral variation (e.g., cm-scale veins, post-depositional concretions) to constrain provenance and diagenetic history.
2. Assess current atmospheric and astronomical conditions, events, and surface-atmosphere interactions and processes	2-a. Observe the Sun for rover navigation and atmospheric science purposes.
	2-b. Observe the sky and surface/atmosphere boundary layer to measure atmospheric aerosol/cloud properties and transient atmospheric/astronomical events.
3. Provide operational support and scientific context for rover navigation, contact science, sample selection, extraction, caching, and other Mars 2020 investigations	3-a. Acquire stereo images for navigation, instrument deployment, and other operational purposes on a tactical timescale.
	3-b. Acquire sub-mm/pixel scale images of targets close to the rover.
	3-c. Resolve morphology and color/multispectral properties of distant geologic features and topography for longer-term science and localization/navigation planning purposes.

The specific high-level goals of the Mastcam-Z investigation are:

*Goal 1: Characterize the overall landscape geomorphology, processes, and the nature of the geologic record (mineralogy, texture, structure, and stratigraphy) at the rover field site.* Mastcam-Z observations will provide data necessary for a full description of the topography, geomorphology, geologic setting, and the nature of past and present geologic processes in Jezero crater, especially as they pertain to habitability. This includes observations of rocks and outcrops to help determine morphology, texture, structure, stratigraphy and stratigraphic sequence, rock type, mineralogy, depositional or erosional history, and any associated diagenetic and weathering characteristics. Meeting this goal also requires observations of fine-grained regolith (soil, aeolian sand and dust) to help evaluate the nature of their physical and chemical alteration, depositional/erosional processes, and stratigraphy, texture, and mineralogy.

*Goal 2: Assess current atmospheric and astronomical conditions, events, and surface-atmosphere interactions and processes.* This will be achieved by observations of clouds, dust-raising events, properties of suspended aerosols (dust, ice crystals), tracking of dust deposition and removal history on calibration targets, astronomical phenomena, and active aeolian transport of fines. This goal also encompasses characterization of potential ice- or frost-related (periglacial) geomorphic features (if present) and the characterization of any seasonal frost or ice and its influence on rocks and fines.

*Goal 3: Provide operational support and scientific context for rover navigation, contact science, sample selection, extraction, and caching, as well as imaging support for other Mars 2020 instruments and rover tools.* Mastcam-Z images will assist rover navigation by enabling the more accurate determination of the location of the Sun and of horizon features, and by providing information pertinent to rover traversability (*e.g.*, nature of distant hazards, higher-resolution terrain meshes, etc.). This goal also includes observations enabling other Mars 2020 science instruments to identify and characterize potential materials to be collected for *in situ* analyses, coring, and caching, or other purposes (*e.g.*, rover hardware monitoring).

### Mastcam-Z Detailed Investigation Goals and Objectives

#### Goal 1: Landscape Geomorphology and Processes (Objectives 1-a Through 1-g)

Mastcam-Z images will detect tonal, textural, and spectroscopic signatures that occur at the millimeter to decameter scale and will contribute to studies of the morphology and texture of rocks and fines; coatings and weathering rinds on rocks and crusts on fines; sedimentary, igneous, and potentially biogenic structures; the stratigraphic placement of rock outcrops and regolith clasts and fines, and their inferred geologic history; and mineralogy and composition of rocks and fines. These properties can be used to assess not only the geologic and climatic history but also potential habitability and biogeological interactions at the Mars 2020 landing site and along the rover’s traverse path (*e.g.*, Mustard et al. 2013; Noffke et al. 2013), and to select the optimal samples for coring and caching, the first stages of a longer-term Mars sample return campaign.

##### Grain Size, Morphology, and Texture

**Fig. 1:**
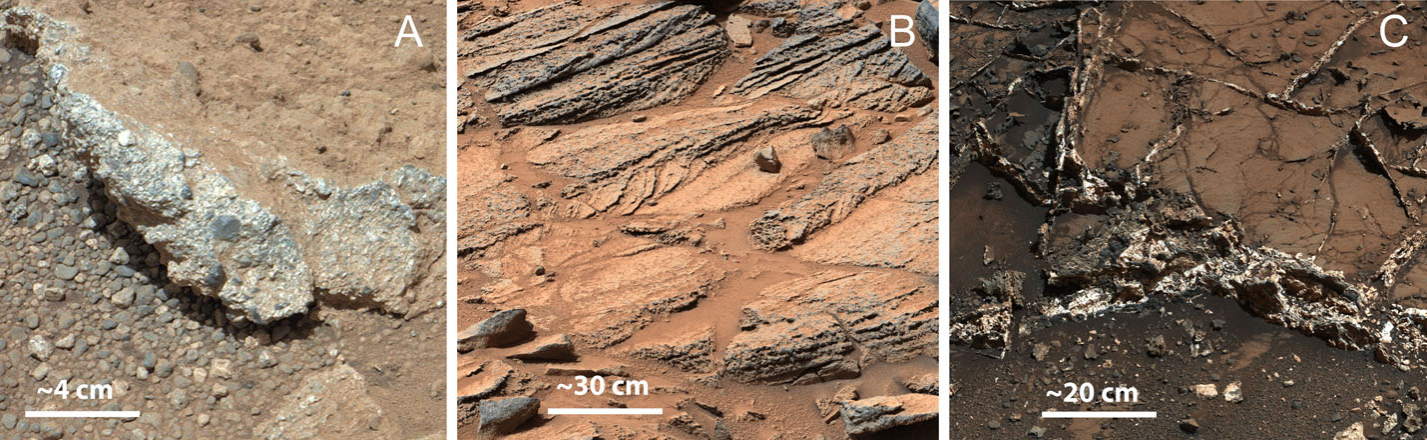
Did water flow on the surface/subsurface of Mars? Like MSL/Mastcam, Mastcam-Z’s resolution can discriminate (**A**) conglomerates formed in high-energy streams (Williams et al. 2013), (**B**) cross-bedded sandstones formed in less energetic flows (Edgar et al. 2018), and (**C**) lake-deposited mudstones with post-depositional circulation of subsurface fluids (Kronyak et al. 2019). These kinds of features provide context in the Mars 2020 search for habitable environments.

Mastcam-Z will document the textural attributes of rocks and fines at granular (hundreds of μm) to outcrop and bedform (cm to m) scales. For example, oriented flutes, pits, scoops, and gouges in rocks often indicate abrasion by wind-driven sand and the direction of the most energetic winds when abrasion occurred (*e.g.*, Sharp 1949; Laity and Bridges 2009; Bridges et al. 2014; Sullivan et al. 2008; Schieber et al. 2020). Observations of spall fragments can indicate expansion/contraction due to thermal, freeze-thaw, or salt weathering processes (*e.g.*, Malin 1976; Anderson and Anderson 2011; Eppes et al. 2015). Clast size and roundness can provide information about fluvial transport velocity and the rapidity of sedimentation, crucial for understanding the potential for biosignature preservation (*e.g.*, Pettijohn 1975; Grotzinger et al. 2005, 2006, 2014; Williams et al. 2013) (Fig. 1). Igneous rock textures, grain sizes, and colors can constrain composition, formation conditions, and volatile content (*e.g.*, McSween et al. 2006a,b). Breccias with poorly sorted angular clasts could indicate pyroclastic origins (fall deposits or density current deposits) or impact processes; other textural observations (*e.g.*, clast vesicularity and lithology) can help to distinguish between volcanic and impact origins (*e.g.*, Cas and Wright 1987).

##### Rock Coatings, Weathering Rinds, and Crusts

**Fig. 2:**
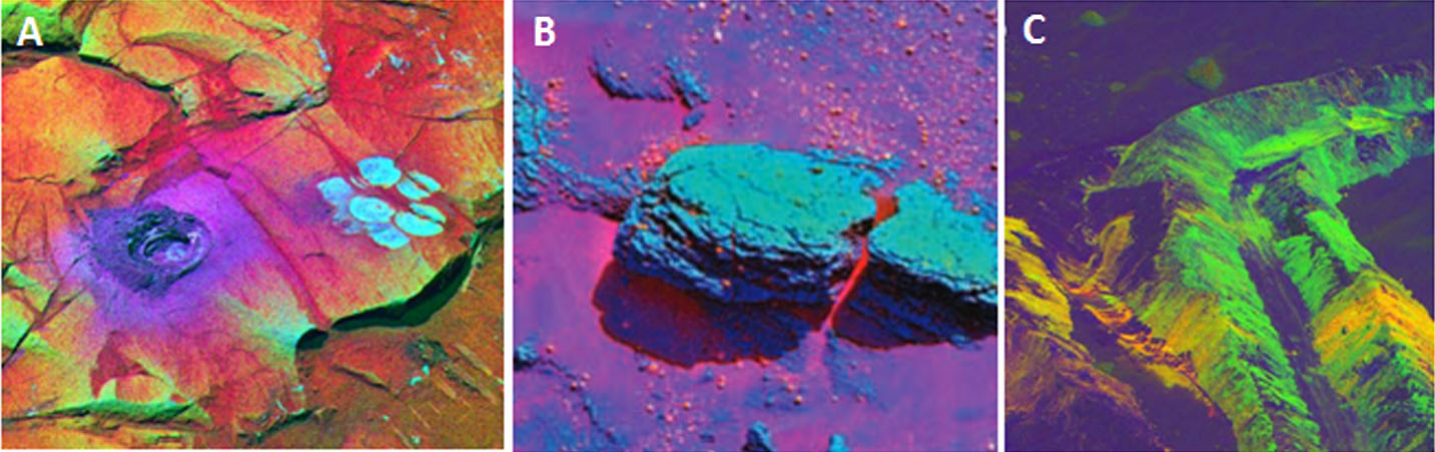
Mastcam-Z’s near-UV, visible, and near-IR filters (Table 3) enable false color composite representations like these to detect (**A**) ferric/ferrous variations in coated vs. abraded surfaces (Farrand et al. 2007); (**B**) hematite in diagenetic concretions (Bell et al. 2004b); (**C**) 1000-nm absorption in hydrated soils in natural or disturbed surfaces (Johnson et al. 2007)

Weathering rinds result from chemical alteration in the presence of water. Based on optical scattering (including glints) and spectral/compositional properties, coatings were inferred on rocks at the *Viking Lander 1*, *Viking Lander 2*, and *Mars Pathfinder* sites (*e.g.*, Guinness et al. 1997; Johnson et al. 1999; Murchie et al. 2004). Evidence for rock coatings was ubiquitous in MER Pancam images of rocks in the Gusev plains (*e.g.*, Bell et al. 2004a; McSween et al. 2006a, 2006b; Johnson et al. 2008; Fig. 2) and in the sedimentary rocks of Meridiani Planum (*e.g.*, Bell et al. 2004b; Farrand et al. 2007, 2008; Weitz et al. 2010) and has also been reported in Gale crater rocks (Ollila et al. 2014; Lanza et al. 2015).

##### Structure

Sedimentary structures in rocks and regolith record atmospheric and geologic processes through a variety of primary features such as bedding, as well as post-depositional deformation of these features via diagenesis, all detectable at Mastcam-Z resolution (*e.g.*, Stow 2005). In subaqueous environments, current ripples indicate higher energy deposition than finer-grained, uniform laminations, and can be used to constrain flow depth and infer aspects of the style and direction of sediment transport (*e.g.*, Herkenhoff et al. 2004; Grotzinger et al. 2005, 2006). Resolving such structures in stereo is particularly important for removing ambiguities caused by geometric effects (*e.g.*, Grotzinger et al. 2006; Hayes et al. 2011). In igneous rocks, structures include a range of features regarding the nature and cooling of lava flows, pyroclastic events, and intrusive processes (*e.g.*, Smith and Katzman 1991; Valentine and Fisher 1999), such as interpreted by the examples imaged by MER/Pancam at Home Plate in Gusev crater (*e.g.*, Squyres et al. 2007; Lewis et al. 2008; Manga et al. 2012).

##### Stratigraphy

Stratified deposits chronicle paleoenvironments and provide glimpses into the broader history of early Mars (*e.g.*, Malin and Edgett 2000; Grotzinger et al. 2005, 2015). Mastcam-Z stereo images and panoramas of the rover’s field site will be crucial for reconstructing the time-series of emplacement events by helping to direct rover *in situ* measurements and sampling to key boundaries in space and time in the rock record. Mastcam-Z’s high-resolution and stereo Digital Terrain Model (DTM) capabilities can be used to map and interpret stratigraphy along the rover’s traverse (*e.g.*, Arvidson et al. 2008; Crumpler et al. 2011; Grotzinger et al. 2014; Stein et al. 2020) and to investigate small-scale layering in fine regolith materials (such as those exposed by wheel trenching; Arvidson et al. 2008, 2011; Sullivan et al. 2008, 2011), and in outcrops to search for evidence of subaqueous deposition (*e.g.*, Grotzinger et al. 2006) and stratigraphic variations in cross-bedding geometries that document paleo-wind directions and other processes (*e.g.*, Hayes et al. 2011; Banham et al. 2018; Barnes et al. 2018).

##### Mineralogy

Mineral identification is a key component of habitability assessment, especially evidence for water, redox gradients or electron donors (*e.g.*, Fe^2+^ vs. Fe^3+^, Mn), or the presence of astrobiologically relevant elements (*e.g.*, Hazen et al. 2008; Summons et al. 2011; Ehrenfreund et al. 2011). Past Mars surface mission experience shows that even low spectral resolution RGB color and multispectral imaging with carefully-selected wavelengths can help investigators decide strategically and tactically along the traverse where to invest in resource-intensive arm-related or other rover activities, and to understand more clearly the context of mineralogic phases identified by other payload elements (*e.g.*, Bell et al. 2008, 2020; Farrand et al. 2008; Blake et al. 2013; Vaniman et al. 2014). In addition, fine-scale compositional imaging can help to constrain the mineralogy of small features. Examples from previous missions include mm- and cm-scale veins (Squyres et al. 2012; Nachon et al. 2017; Kronyak et al. 2019) and mm-scale diagenetic ridges seen by Curiosity at Yellowknife Bay (Grotzinger et al. 2014). Thus, while Mastcam-Z is not a spectrometer, the multispectral capability of Mastcam-Z still addresses important mineralogic context requirements.

**Fig. 3:**
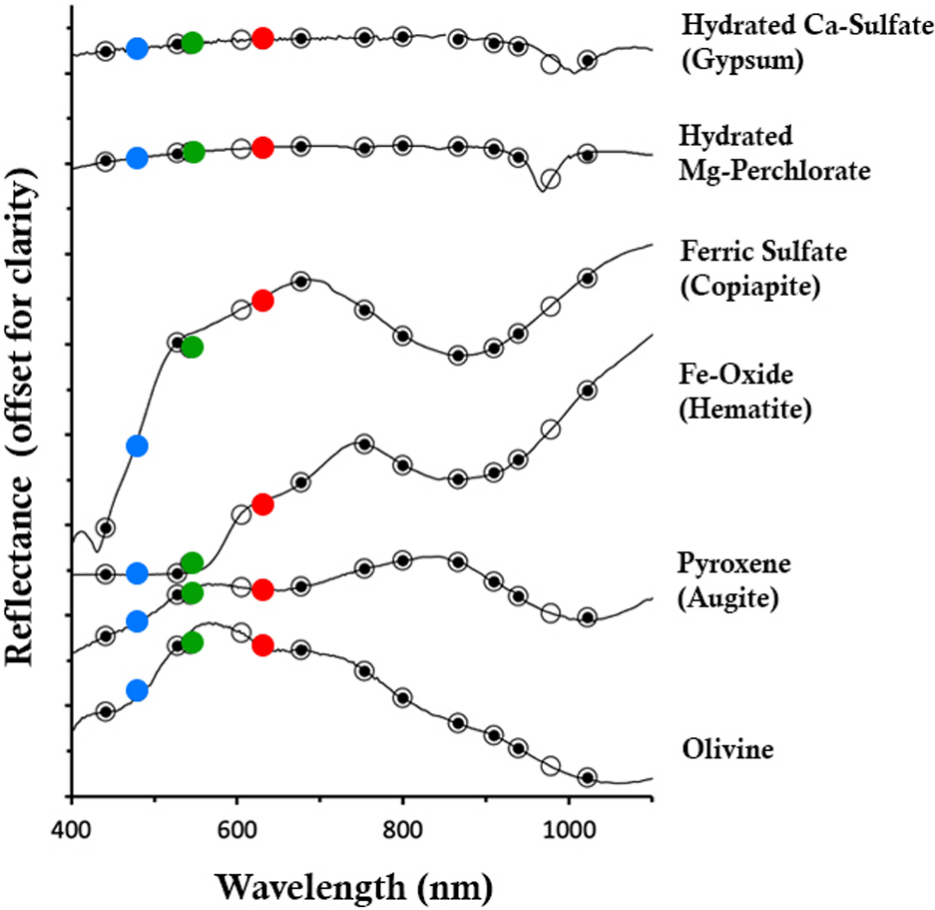
Lab spectra (solid line; Clark et al. 2007) of Mars-relevant hydrated minerals, ferric sulfate, iron oxide, and ferrous silicates convolved to the Mastcam-Z narrowband and RGB filters (open circles). Black circles: heritage MSL/Mastcam filters (Bell et al. 2017)

Specifically, as with previous science imaging systems on Mars Pathfinder, MER, Phoenix, and MSL, the Mars 2020 Mastcam-Z will use narrowband filters (Table 3) to provide low spectral resolution sampling of materials in up to 11 distinct wavelengths (plus broadband RGB color) in the ∼400–1000 nm range. Relatively broad mineral spectral signatures in this range (Fig. 3) include those due to important Fe^2+^-bearing silicates such as high and/or low calcium pyroxenes and olivine in relatively unaltered rocks and fines (*e.g.*, Adams 1975; Cloutis and Gaffey 1991; Bibring et al. 2005; Mustard et al. 2005; Clenet et al. 2013). Additional mineral features observed in this range are characteristic of Fe^3+^-bearing alteration products, including a number of specific iron oxides, oxyhydroxides, phyllosilicates, and oxyhydroxysulfates (*e.g.*, Morris et al. 1985), as well as some hydrated minerals (*e.g.*, silica, H_2_O ice, some sulfates, phyllosilicates, carbonates and hydrated perchlorates) that show a narrower and weak H_2_O and/or OH absorption feature near 950–1000 nm (Wang et al. 2008; Rice et al. 2010). While many of the filters used for Mastcam-Z are close to or essentially the same as those used for MER/Pancam and MSL/Mastcam as well as those planned for the PanCam instrument on the 2022 ExoMars *Rosalind Franklin* rover (Coates et al. 2017), the Mastcam-Z filter set additionally features different positioning of several narrowband filters that, based on laboratory data as well as MER and MSL experience, could enable better detection of hydrated minerals and iron-bearing phases than previously used filters (*e.g.*, Rice et al. 2010, 2020; Gunn and Cousins 2016), as well as better potential detectability of the mineral diversity specifically expected in and around Jezero crater, as detected from orbit (*e.g.*, Ehlmann et al. 2008, 2009; Murchie et al. 2009; Horgan et al. 2020). Mastcam-Z will also address mineralogy indirectly through investigations of shape, cleavage, color, and luster of mineral grains and fragments (*e.g.*, Herkenhoff et al. 2004, 2019; Bell et al. 2008; Yingst et al. 2013). Collectively, Mastcam-Z’s RGB and narrowband filters should allow certain compositional variations to be rapidly mapped along the rover traverse. This will help direct the science team’s tactical and strategic attention to the most valuable locations for sampling activities that will inform most effectively about the history of Mars, including ancient aqueous, potentially habitable environments.

**Table 3:**
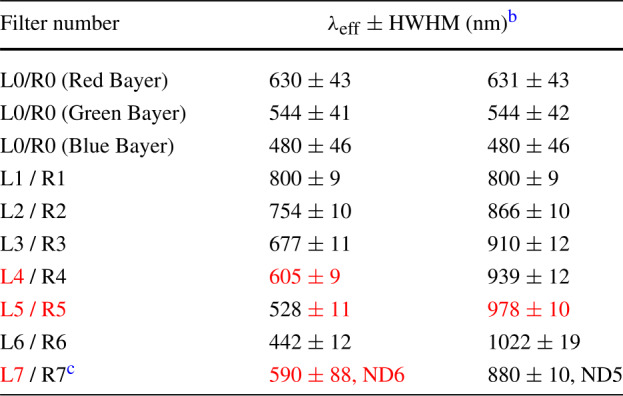
Mastcam-Z Left (L) and Right (R) filters^a^

^a^Red text means new performance compared to MSL/Mastcam.

^b^*λ*_eff_ is the effective band center wavelength, calculated as the weighted average of the normalized system spectral response (including optics, filter, and CCD) and the solar radiance at the top of the Martian atmosphere. HWHM is the half-width of the bandpass at half-maximum for each filter.

^c^Filters L7 and R7 are for direct imaging of the Sun using Neutral Density (ND) coatings that attenuate the flux by factors of 10^6^ (ND6) and 10^5^ (ND5), respectively. Filter L7 enables 3-color (RGB) Bayer filter color imaging of the Sun at the same effective band center wavelengths as the L0 and R0 filters.

See Hayes et al. (2021) for details on these derived values.

#### Goal 2: Atmospheric, Meteorological, and Astronomical Observations (Objectives 2-a,b)

Mastcam-Z will directly characterize multiple aspects of atmospheric aerosols to understand the current climate of Mars and potential hazards for future exploration (Tables 1 and 2). For example, Mastcam-Z’s direct solar imaging capability (with Neutral Density filters sampling four different VNIR colors: RGB+880 nm; Table 3) offers the most accurate method available for measuring column-integrated aerosol abundances (*e.g.*, Smith and Lemmon 1999; Lemmon et al. 2004, 2015, 2019). These opacity data will be combined with the high radiometric fidelity of (diffuse) sky observations to constrain the particle size and other macrophysical properties of the dust (*e.g.*, single scattering albedo; Pollack et al. 1995; Tomasko et al. 1999; Lemmon et al. 2004; Bell et al. 2006a; Wolff et al. 2006). In addition, Mastcam-Z will extend the record of continuous ground-truth observations dating back to 2004 that are leveraged for planet-wide, orbiter-based studies (*e.g.*, Wolff et al. 2009, 2010, 2019; Tamppari et al. 2010). Direct solar imaging will also enable occasional observations of solar transits of Phobos and Deimos, improving knowledge of their ephemerides and physical properties (*e.g.*, Bell et al. 2005; Lainey et al. 2007; Jacobson 2010; Lemmon et al. 2013). Twilight or night-time imaging also offers opportunities for extending knowledge of dust optical depth values to a full diurnal cycle through imaging of stars or other astronomical targets, as well as the potential for increasing our knowledge of the present-day meteoroid flux at Mars (*e.g.*, Domokos et al. 2007).

Local sources of dust will be investigated through dust devil monitoring (*e.g.*, Ferri et al. 2003; Greeley et al. 2010), and the rate and properties of dust sedimentation on the rover will be tracked with rover deck calibration target and other monitoring (*e.g.*, Landis and Jenkins 2000; Kinch et al. 2015, 2020a; Drube et al. 2010; Vicente-Retortillo et al. 2018), producing a temporal record of aerosol properties that can affect both surface and atmospheric heating rates and thus atmospheric dynamics (*e.g.*, Fenton et al. 2007; Madeleine et al. 2011). Additionally, sky imaging of water and/or CO_2_ ice clouds can constrain microphysical and dynamical models (*e.g.*, Colaprete et al. 2003; Madeleine et al. 2012; Wolff et al. 2017, 2019). Finally, we will investigate the magnitude of modern surface-atmosphere interactions (*e.g.*, hydration, desiccation) by monitoring tonal, color, and spectroscopic changes at locations where the rover has freshly exposed rock interiors or subsurface materials (*e.g.*, Wang and Ling 2011; Rice et al. 2011, 2013; Wang et al. 2013), comparing them to laboratory experiments (*e.g.*, Cloutis et al. 2008; Altheide et al. 2009).

#### Goal 3: Mastcam-Z Operational Support (Objectives 3a Through 3-c)

As summarized in Tables 1 and 2, the Mastcam-Z investigation also includes significant operational support objectives. These include high-fidelity and rapidly-processed images, mosaics, and other derived products (*e.g.*, DTMs and videos) designed to support rover driving decisions, dust removal, abrasion, and coring tool placement, contact science instrument positioning, and assessment/anomaly diagnostics on the state of rover components visible to either or both Mastcam-Z cameras. Past experience from Mars landers and rovers provides ample examples of the ways that high-resolution and/or multispectral imaging capabilities can enhance the operational efficiency or fidelity of surface missions. For example, the science cameras on the Mars Pathfinder lander (Smith et al. 1997) and on the MER *Spirit* and *Opportunity* rovers (Bell et al. 2006b; Herkenhoff et al. 2019) frequently provided tactically-useful higher-resolution and color imaging data for rover navigation and instrument targeting decisions, augmenting information available from lower resolution and monochrome rover engineering cameras. Similar support by the Mastcam instruments has augmented engineering camera (Hazcams and Navcams) observations on the MSL *Curiosity* rover as well (Maki et al. 2012; Malin et al. 2017). On *Perseverance*, the engineering cameras will have RGB color capability and the potential for higher spatial resolution than the engineering cameras on *Curiosity* (Maki et al. 2020). Regardless, we anticipate that imaging from Mastcam-Z will be used to substantially enhance the information needed for rover driving or engineering-related decisions because its resolution and color capabilities still exceed those of the upgraded *Perseverance* engineering cameras.

### Design Considerations, Requirements, and Heritage

High-resolution panoramic, stereoscopic, and color/multispectral context images are among the measurement types generally agreed by the Mars science community to constitute threshold requirements to efficiently characterize the geology of a site, to assess its past habitability, to search for biosignatures, and to assist with sample caching (including detailed assessment of the context of cached samples) and strategic knowledge gap (SKG) filling activities (*e.g.*, Mustard et al. 2013). While multispectral imagers alone cannot meet all of those community-identified requirements, their data can contribute significantly to geologic characterization, the characterization of some potential biosignatures, and planning and implementation of operational activities like sample caching. In addition, data from multispectral imagers can provide significant support and guidance to other instrument investigations that are specifically focused on meeting habitability and SKG-filling goals.

In Table 4, we trace the connections between Mastcam-Z investigation goals and objectives (Tables 1 and 2) to a subsequent set of observational requirements and instrument functional requirements that enable the system to meet both the investigation and relevant mission requirements. To facilitate tactical relevance, our geologic and operational observing objectives in Table 4 are separated into near-field (∼1 to 5 m range) and mid-field (∼5 to 100 m) based on past mission experience and expected typical rover drive distances. Atmospheric and astronomical objectives will be met with predominantly far-field observations (>100 m to infinity).

**Table 4:** Mars 2020 Mastcam-Z science traceability to observational and functional requirements

Mastcam-Z goals^a^	Mastcam-Z objectives^b^		Observation requirements	Instrument functional requirements
1. Characterize the overall landscape geomorphology, processes, and the nature of the geologic record (mineralogy, texture, structure, and stratigraphy) at the rover landing site and along the rover’s traverse	Mid-field ∼5 to 100 m away	1-a	• Acquire color monoscopic or stereo images and panoramas of extended regions around the rover under near-constant illumination conditions • Acquire 360° contextual panoramas at key locations along the traverse to document geologic context and to assess stratigraphic boundaries • Tactically assemble DTMs • Resolve cm-size bedding planes, contact geometries, rocks, veins, and nodules up to 60 m from the rover • Spectrally discriminate among expected Mars surface materials to help assess redox state and to choose best *in situ* and coring targets	• Cameras must be mounted as a stereo pair capable of near-simultaneous (<1 s) imaging • System must have an Instantaneous Field of View (IFOV) of ≤75 μrad/pix at full zoom (high resolution) and ≤3 mrad/pixel at wide angle (low-resolution) • System must be able to buffer ≥1 Gbit of data within internal (non-rover) Non-Volatile Random Access Memory (NVRAM) • System must be capable of imaging at any time during the day or night • Cameras must have RGB color imaging and high resolution bandpass filters to interrogate the ferric absorption edge (< 600 nm), ferric/ferrous spectral slopes/bands in the near-infrared (700-1000 nm), and near-IR hydration band (965 nm) *Relevant Requirements on Remote Sensing Mast* • Cameras must be able to be pointed to enable 360° azimuth coverage and −90° to +90° elevation coverage • System must be able to acquire a 360° panorama of the surrounding terrain at MER/Pancam scale or better (≤273 μrad/pix) in <1 hour
		1-b		
		1-c		
		1-d		
	Near-field ∼1 to 5 m away	1-e	• Acquire individual color monoscopic or stereo images that can resolve mm-size bedding planes, contact geometries, rocks, veins, and nodules within about 1.5 to 4 meters of the rover • Repeat images of a scene at multiple illumination angles for photometric studies	
		1-f		
		1-g	• Use RGB color images to discriminate visual color differences of bulk materials • Use narrowband color filter images to detect and discriminate among major Fe-bearing silicates, ferric oxides, ferric oxyhydroxides, and selected hydrated and/or hydroxylated minerals and to identify cm- to dm-scale signs of alteration in outcrop	• Image through specific RGB and narrowband (10-20 nm width) filters in specific visible to near-infrared (400-1000 nm) wavelengths (Table 3) that enable dust cover to be assessed and diagnostic Fe-bearing and hydrated and/or hydroxylated minerals to be detected and distinguished
2. Assess current atmospheric and astronomical conditions, events, and surface-atmosphere interactions and processes	2-a		• Image the Sun in at least 2 colors sufficient to distinguish atmospheric dust and water ice aerosols	• Acquire direct images of the Sun near 440 and 880 nm using neutral density (ND) filters
	2-b		• Acquire images of the sky at wavelengths that constrain aerosol physical and radiative properties • Use camera temp. sensors to estimate environmental temperature at camera height above ground	• Use narrowband filters near 440 and 880 nm to ≈ match solar filters, span ≈2x in wavelength, and include low and high band wavelengths • Acquire temperature measurements with an accuracy of ±2 °C using instrument temp. sensors
			• Acquire rapid (≥2 fps) ≤2 min videos with ≥ MSL-M100 resolution over 5° FOV for transits; and ≤10 min color videos at ≥1/3 fps with at least Pancam resolution and FOV for dust devils and clouds	• RGB high-definition video at a rate of at least 2 frames/second
3. Provide operational support and scientific context for rover navigation, contact science, sample selection, extraction, and caching, and other selected Mars 2020 investigations	Mid-field ∼5 −100 m	3-a	• Acquire stereo images, mosaics, or panoramas of extended regions around the rover under near-constant illumination conditions • Acquire images of rover hardware and arm workspace at varying spatial scales sufficient to assess configuration, dustiness, basic wear, etc.	• Resolve driving hazards comparable to the size of a rover wheel radius (25 cm) at 100 m • Generate near-field terrain meshes with a range error of <5 mm • Acquire in-focus images that resolve features from ≤3 mm size at 1.5 m (calibration target, deck) to ≤10 cm size at 100 m range
		3-b	• Resolve (identify and characterize) rocks or other potential rover driving obstacles as well as potential fiducials for locations from orbiters	
	Near-field ∼1.5-5 m	3-c	• Acquire multispectral (color-contrasting) images of soils, clasts, and rocks/outcrop close to the rover under relatively high-Sun illumination	• Image in specific RGB and narrow (10-20 nm) wavelengths that enable assessment of dust cover and diagnostic Fe-bearing and hydrated minerals to be detected and distinguished
			• Resolve medium to coarse sand-sized grains and clasts in the in-situ instrument work volume, providing context for fine-scale imaging investigation and selection of samples for caching • Acquire images at varying spatial scales	• Acquire in-focus images at a pixel scale of 150 μm/pix at 2 m range • System must be able to acquire images at varying spatial resolutions

^a^See Sect. 2.1 for detailed definitions of Mastcam-Z Investigation Goals.

^b^See Table 2 for detailed definitions of Mastcam-Z Investigation Objectives.

As outlined in Table 4, Mastcam-Z must be able to provide RGB color panoramic capability, sufficient resolution in the far-field (resolving ∼3-4 cm features at 100 m) and in the near field (resolving ∼1 mm features in the arm’s work volume in front of the rover), and visible to short-wave near-IR (∼400–1000 nm) multispectral capability to distinguish more dusty from less/not dusty materials and to provide important insights into the mineralogy of iron-bearing silicates, oxides, and oxyhydroxides, as well as some classes of diagnostic hydrated minerals. Mastcam-Z images are required to have a Signal-to-Noise Ratio (SNR) of at least 30:1 for all filters at an exposure duration of 0.015 seconds for low albedo (0.1 in red) targets at high solar incidence (80°) at aphelion on Mars. The Modulation Transfer Function (MTF), which incorporates contrast and resolution to determine the total resolution performance of the camera, is required (as on MSL/Mastcam) to be >0.2 at the maximum sampling (Nyquist) frequency for the entire camera system (optics + filters + CCD).

In addition, Mastcam-Z must have improved stereo imaging capabilities compared to the MSL/Mastcams and MER/Pancams to be able to substantially augment the navigational and instrument placement capabilities of the *Perseverance* engineering cameras and to support and enhance Mars 2020 driving and coring/sampling capabilities. Specifically, Mastcam-Z DTMs must be able to resolve driving hazards at ranges several times greater than nominal Navcam data allow, and facilitate finer-position planning of arm deployment for contact science than nominally capable using Navcam or Hazcam data. Mastcam-Z DTMs thus are designed to exceed the nominally recommended range resolution of 1 mm at 2 m range, or a resolution of 2 cm at 10 m distance (*e.g.*, Mustard et al. 2013). Finally, to support expected Mars 2020 operational timelines, based on MSL/Mastcam experience and heritage, Mastcam-Z must be able to acquire RGB color 360° × 70° wide angle (26 mm focal length; 0.28 mrad/pix) panoramas in ≤1 hour, and downlink those data in ≤2 sols. As described below and in Hayes et al. (2021), the as-built Mastcam-Z cameras exceed these requirements by healthy margins.

## Instrument Description

**Fig. 4:**
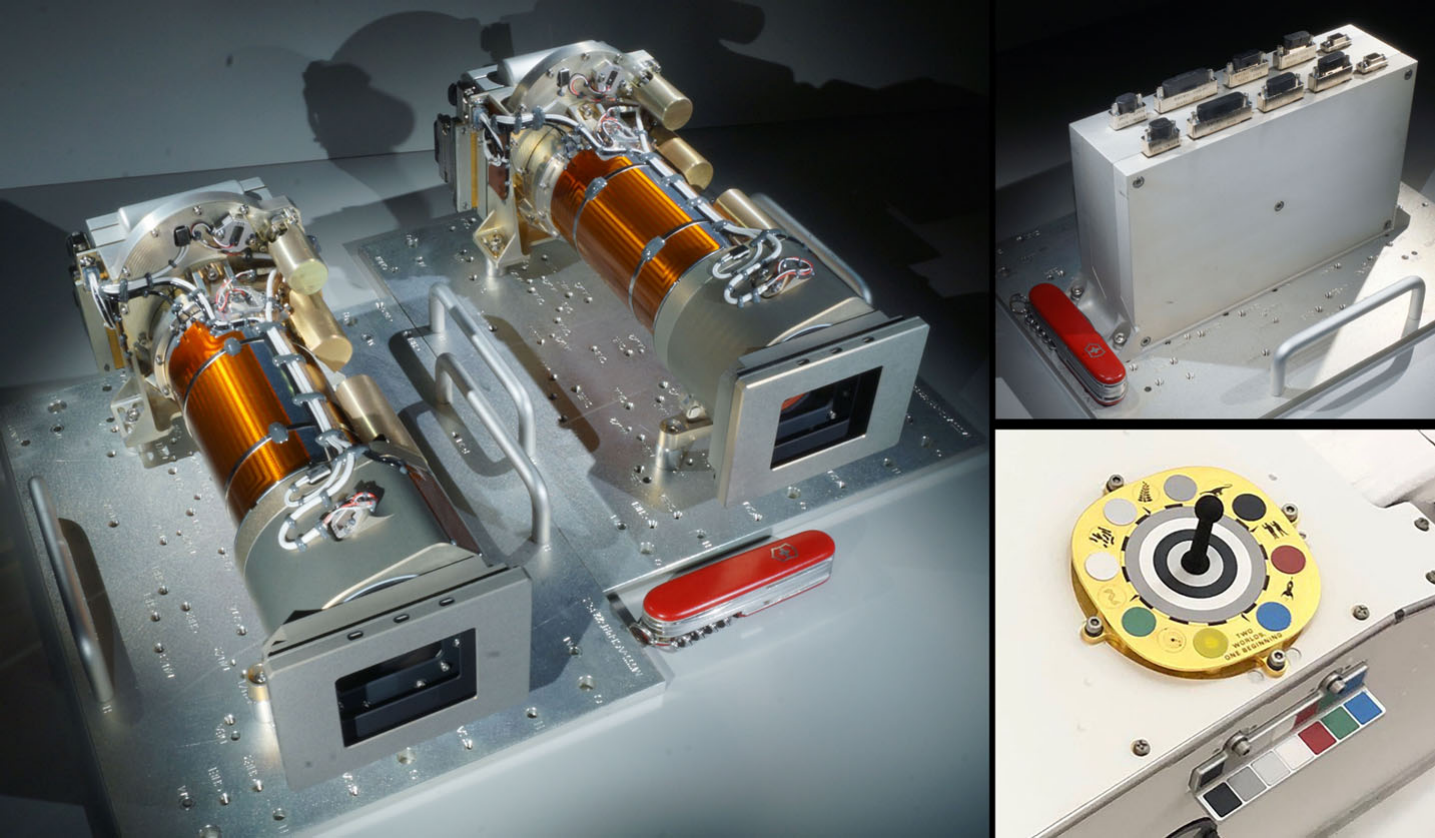
Mars 2020 *Perseverance* rover Mastcam-Z flight hardware. (Left) Flight Camera Heads (identical to each other except for different color filters in their filter wheels; Table 3), shown upside-down on temporary carrying plates. (Upper right) Flight Digital Electronics Assembly (DEA), on a temporary carrying plate. For scale, the pocket knife in the photos is 3.5 inches (88.9 mm) long. (Lower right) Flight Primary (top) and Secondary (lower) Calibration Targets, mounted on the rover deck. For scale, the primary target is 3.1 × 3.1 inches ($8 \times 8$ cm) across

The Mars 2020 rover Mastcam-Z flight hardware consists of 5 elements (Fig. 4): two camera heads, mounted on the camera plate on the rover’s Remote Sensing Mast (RSM); one Digital Electronics Assembly (DEA) with two electronics cards (one per camera head) mounted into a single housing located inside the rover chassis, and two passive calibration targets, mounted on top of the Rover Pyro Firing Assembly (RPFA) box on the rover deck, close to the same location used for the *Curiosity* Mastcam calibration target (Bell et al. 2017). The Mastcam-Z camera heads each consist of an optomechanical lens assembly (with focus and zoom actuators), a filter wheel (with actuator), and a focal plane assembly and its electronics. Table 5 summarizes the Mastcam-Z instrument characteristics.

**Table 5:** Mars 2020 *Perseverance* rover Mastcam-Z instrument characteristics

Optics	Description	
Focus	Adjustable; Working distances 0.5–1.0 m to ∞	
MTF	>0.35 at Nyquist (optics + filters + CCD)	
Filter bandpasses	Two 8-position filter wheels: see Table 3	
*Zoom-Dependent:*	*Widest Field *	*Narrowest Field*
FOV (1600 × 1200 pix)	25.6° × 19.2°	6.2° × 4.6°
IFOV	283 μrad	67.4 μrad
Focal ratio	*f*/6.7	*f*/9.5
Effective focal length	26 mm	110 mm
*Detector & Electronics*	*Description*	
CCD	ON Semi (Kodak) KAI-2020CM interline transfer	
Color	Red, Green, Blue microfilters, Bayer pattern	
Array size	1600 × 1200 photoactive pixels (1648 × 1214 total)	
Pixel size	7.4 μm (square pixels)	
Gain, Read Noise, Full Well	15.6/15.6 e$^{\text{--}}$/DN; 22/21 e$^{\text{--}}$; 21826/21818 e$^{\text{--}}$ (Left/Right)	
Digitization	11 bits/pixel; single gain, no offset states	
Data Interface	Synchronous LVDS: 8 Mbit/sec	
Command Interface	2 Mbit/sec serial link	
Memory	128 MB SDRAM and 8 GB flash buffer for each camera	
Power	7.5 W standby and 11.8 W imaging, per camera	
*Exposure*	*Description*	
Duration	0 to 838.8 sec; commanded in units of 0.1 msec	
Auto-exposure	Based on MSL and MER auto-exposure algorithm (Maki et al. 2003)	
*Onboard Compression*	*Description*	
Uncompressed	11-bit data; No compression; No color interpolation	
Lossless	∼1.7:1 lossless compression; no color interpolation	
Lossy	Realtime JPEG; color interpolation or grayscale; commandable color subsampling Y:C_R_:C_B_ (4:4:4 or 4:2:2) and compression quality (1–100)	
Video Group of Pictures (GOP)	JPEG-compressed color-interpolated GOPs, ≤2 MB file size and ≤16 frames/GOP; commandable color subsampling and compression quality	
Deferred compression	Image can be stored onboard the Mastcam-Z DEA uncompressed; specified compression can be performed at a later time for transmission to Earth	
Z-Stacking for focus merges, range mapping	Reduce as many as eight 1600 × 1200 raw images to a single 1600 × 1200 best-focus color JPEG plus a grayscale JPEG range image	
Companding	11-bit to 8-bit square-root encoding/decoding via lookup tables	
*Physical*	*Description*	
DEA Dimensions	22 × 12 × 5 cm	
Camera Head	11 × 12 × 26 cm (each camera)	
Primary Cal Target	98 × 98 × 8 mm base, plus 37.5 mm high gnomon	
Secondary Cal Target	80 × 30 × 16 mm (width × height × depth)	
Stereo Baseline	24.4 cm; toe-in angle between cameras: 2.3°	
Mass: Camera Head	1.38 kg each	
Mass: DEA	1.47 kg	
Mass: Cal Targets	Primary: 103 g; Secondary: 15 g	

### Focal Plane Array and Electronics

**Fig. 5:**
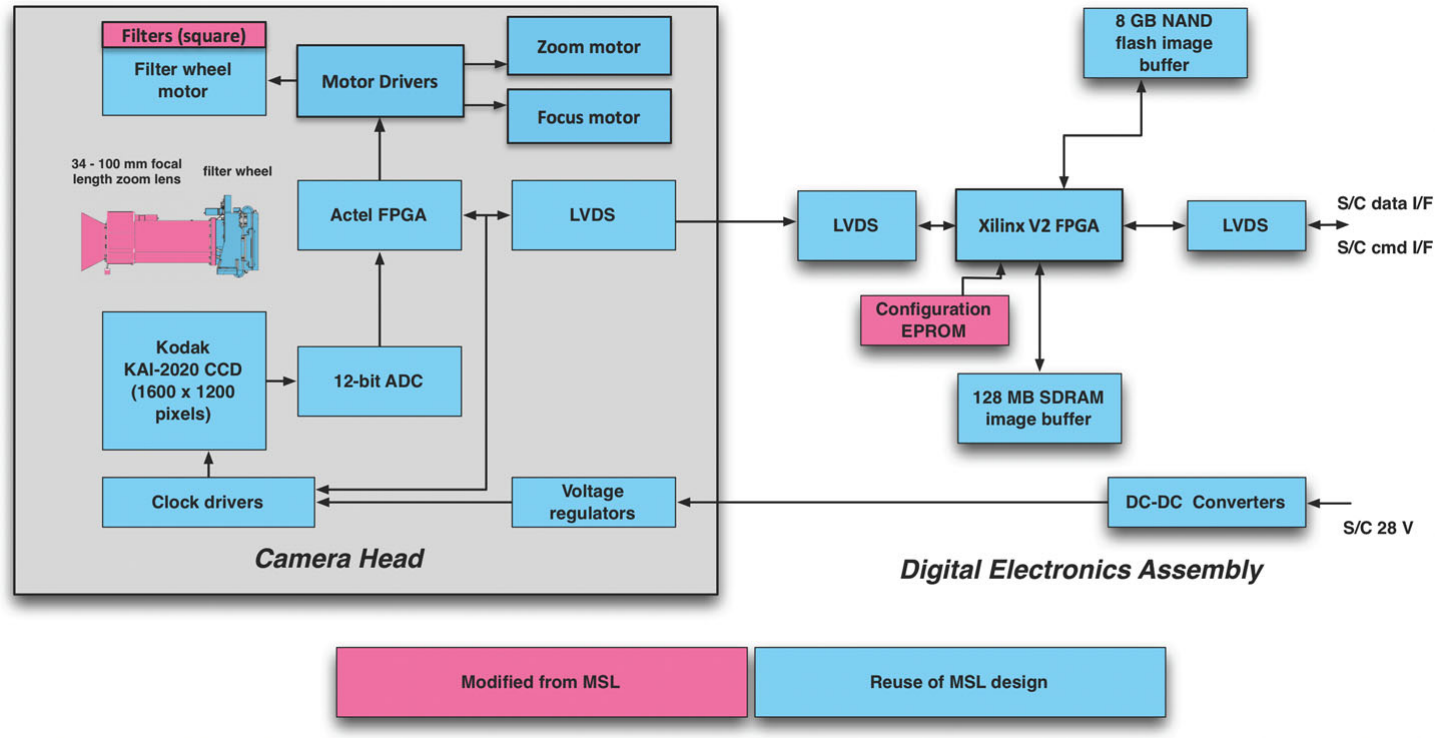
Mastcam-Z Camera Head (Left) and DEA (Right) block diagram.

The Mastcam-Z focal plane array (FPA) and electronics (Fig. 5) are essentially build-to-print copies of the heritage MSL Mastcam FPA (Malin et al. 2017); only obsolescent parts have been replaced with modern equivalents. The Mastcam-Z FPA is designed around an ON Semiconductor (formerly Truesense Imaging, and before that, Kodak) KAI-2020CM interline transfer CCD sensor. Details of the sensor, electronics, and timing signals for the FPA are identical to those described for MSL Mastcam (Malin et al. 2017; Bell et al. 2017) and are thus only summarized here. The sensor has 1600 × 1200 photoactive pixels of 7.4 × 7.4 μm size with no cover glass but with superimposed red, green, blue (RGB) filtered microlenses arranged in a Bayer pattern (see Hayes et al. 2021 for details). The microlenses improve detector quantum efficiency, which has a peak of ∼40% in the red, green, and blue Bayer filters. The output from the CCD is AC-coupled, amplified, and digitized by an analog-to-digital converter (ADC) at a maximum rate of 10 Mpix/s. For each pixel, both reset and video levels are digitized and then subtracted in the digital domain to perform Correlated Double Sampling (CDS), resulting in 11-bits of dynamic range (raw image Data Number (DN) values of 0-2047).

The camera head electronics are laid out as a single 3-section rigid-flex printed circuit board, with sections sandwiched between housings that provide mechanical support and radiation shielding; the interconnecting flexible cables are enclosed in metal covers. Camera head functions are supervised by a single Actel RTSX Field-Programmable Gate Array (FPGA). In response to commands from the DEA, the FPGA generates the CCD clocks, reads samples from the ADC and performs digital Correlated Double-Sampling (CDS), and transmits the pixels to the DEA. The FPGA is also responsible for operating the small brushless DC stepper motors that drive the focus, zoom, and filter wheel mechanisms. The camera head uses regulated +5 V and ±15 V power provided by the DEA. A Platinum Resistance Thermometer (PRT) on the electronic board near the CCD provides temperature knowledge for radiometric calibration. An additional pair of PRTs and redundant etched-foil Kapton heaters are attached to the outside of the camera head and thermostatically controlled by the rover to warm the mechanism for operation when needed. On Mars, the camera head temperatures will be restricted to be nominally operated at temperatures of −40 °C to +40 °C. The heaters can draw up to 72 W of power to bring the cameras above their operational allowable flight temperature from the worst-case (nighttime) cold conditions in just over 1 hour. This design has been verified during pre-flight testing to be able to survive more than two Mars years of diurnal temperature cycles (down to –130 °C) without any heating issues. Performance has also been verified by (as of this writing) nearly 3000 sols (more than 8 Earth years) of successful operation of the *Curiosity* Mastcam instruments on Mars.

### Optomechanical Design

**Fig. 6:**
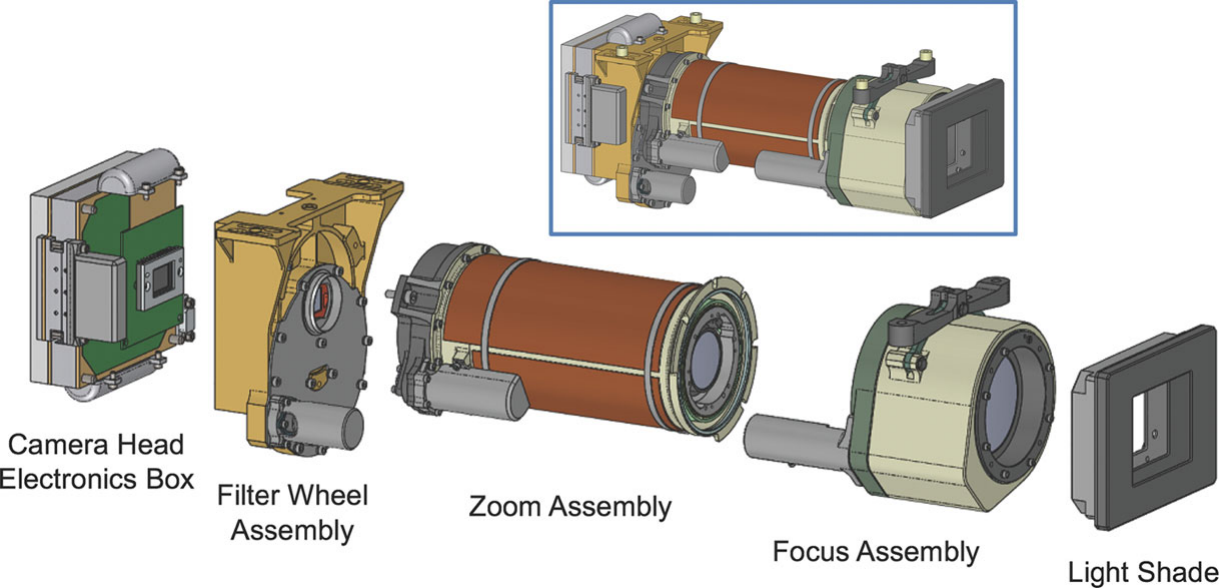
CAD model renderings of the Mastcam-Z optomechanical subsystems. Inset shows the full Camera Head.

The optomechanical assembly for each Mastcam-Z camera head consists of an electronics box, filter wheel, zoom lens, and focus mechanism subsystem assemblies, plus a short light shade with baffles to mitigate stray light (Fig. 6). Two independent cameras enable stereo imaging and block redundancy on the zoom capability. The design is a modification of the focus/zoom/filter wheel assembly that had originally been proposed for MSL Mastcam, but which was ultimately descoped to the two fixed-focal length but variable-focus cameras actually flown on *Curiosity* to Mars (Malin et al. 2017). For Mastcam-Z, a zoom design with a more limited range and less stringent tolerances was fabricated. Specifically, the zoom range has been reduced from the ∼16:1 (6.2 mm to 100.4 mm focal length) range of the originally proposed MSL zoom (DiBiase et al. 2012) to ∼4:1 (26 mm to 110 mm), meeting spatial resolution requirements while simplifying the optical design, reducing the sensitivity to positional variances of the lens elements, and greatly reducing the risks of problems during the manufacturing and assembly of the optics.

**Fig. 7:**
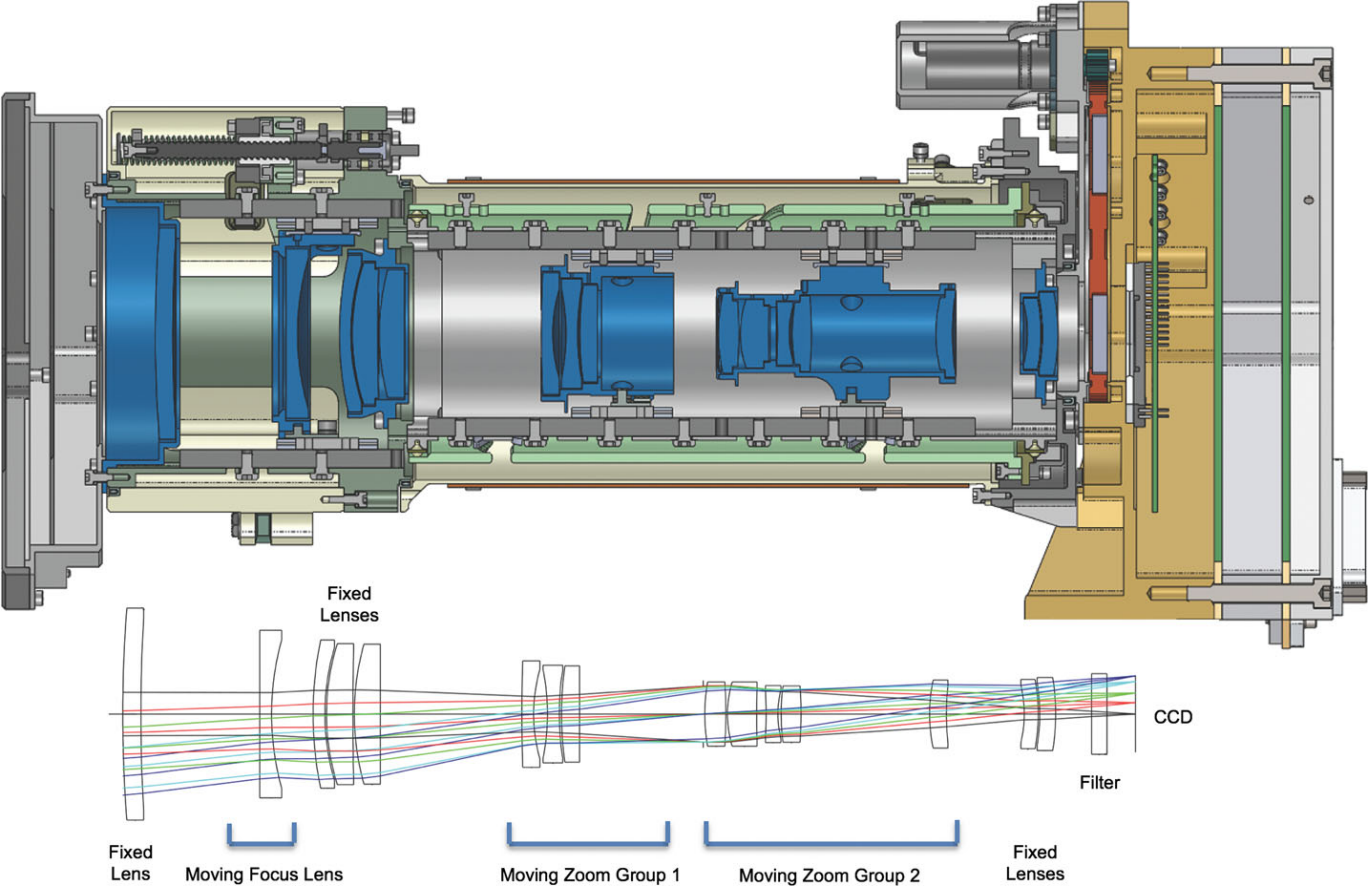
(Top) Cross-sectional view of the Mastcam-Z Camera Head and optics; (Bottom) Ray trace of the Mastcam-Z optics. The movable Zoom Groups and movable Focus Lens are shown here as set at a mid-range focal length of ∼64 mm

The zoom is an all-refractive design consisting of one moving focus group and two moving zoom groups, three stationary groups, and a plano element (the filters) (Fig. 7). The lens can focus as close as 0.5 m at focal lengths of 26 to ∼50 mm and as close as 1.0 m for focal lengths of ∼50 to 110 mm. The focal ratio ranges from $f$/9.5 at 110 mm down to $f$/6.7 at 26 mm. The optics design itself has an MTF of >0.4 at the Nyquist frequency of the detector (68 l.p./mm) over the range of focal lengths and from wavelengths of 400–1000 nm, and the overall camera system MTF (including filters and detector) is >0.35, exceeding the >0.2 requirement.

The optics and all moving parts are sealed within the optomechanical lens housing to prevent dust contamination. Three Cobham (formerly Aeroflex) 10 mm stepper motors drive the Mastcam-Z mechanisms: one each for the focus, zoom, and filter wheel. The focus motor drives a lead screw to provide a 9 mm range of travel for the focus group. The zoom motor drives the integral gear of a cam tube, driving the two zoom groups along linear bearings under the control of cam follower pins. For both of these mechanisms, end-of-travel sensing is provided by a Hall-effect sensor and magnet pairs on the moving component. The filter wheel motor uses a pinion-spur arrangement to drive the outer circumference of the filter wheel, which rides on an integral bearing along its inner circumference. Each of these mechanisms was qualified on MSL, via pre-flight life testing and, for the focus and filter wheel mechanisms, by their actual performance on Mars.

Each filter wheel has eight filter positions (Table 3). The only modifications of the filter wheel from MSL are that the circular, 12.6 mm diameter filters used for those cameras have been replaced with rectangular 13.4 × 10.1 mm filters (to make full use of the rectangular CCD’s field of view), several of the filters have slightly different bandpass characteristics (see Sect. 3.4), and the near-IR stereo imaging wavelength has been changed to 800 nm to provide increased SNR. Otherwise, the filter wheel components are build-to-print copies of the MSL Mastcam design.

### Digital Electronics Assembly (DEA)

The DEA is essentially a copy of the DEA built for the MSL Mastcams (Malin et al. 2017), but with a smaller aluminum enclosure that packages just the two Mastcam-Z Printed Circuit Boards (PCBs), rather than the four PCBs packaged in the DEA housing for the four MSL science cameras (*i.e.*, Edgett et al. 2012; Malin et al. 2017).

#### DEA Hardware

The DEA electronics are laid out on a single rectangular PCB per camera head. The DEA interfaces the camera head with the *Perseverance* rover avionics, which are very similar to the avionics on the *Curiosity* rover. All data interfaces are synchronous (dedicated clock and sync signals) and use low-voltage differential signaling (LVDS). Each (redundant) rover interface comprises two flow-controlled serial links, one running at 2 Mb/s from the rover to the DEA and another at 8 Mb/s from the DEA to the rover. The DEA transmits commands to the camera heads using a 2 Mb/s serial link and receives image data from the camera heads on a 30/60/120 Mb/s selectable aggregate throughput 6-bit parallel link. The DEA is powered from the rover’s +28 V power bus and provides switched regulated power to each camera head. Each PCB also contains a PRT for temperature monitoring. The core functionality of the DEA is implemented in a Xilinx Virtex-2 FPGA. All interface, compression, and timing functions are implemented as logic peripherals of a Microblaze soft-processor core in the FPGA. The DEA provides an onboard image-processing pipeline that includes 11-to-8-bit companding (a term for compression, then later expanding; *e.g.*, Bell et al. 2017), horizontal sub-framing, and optionally lossless predictive (Huffman-encoded; Malin et al. 2013) or lossy JPEG compression. The latter also requires the Bayer pattern raw image to be interpolated and reordered into luminance/chrominance block format. The JPEG interpolation scheme used for Mastcam-Z is identical to the scheme used for MSL/Mastcam (described in Sect. 5.2.1 of Bell et al. 2017), except for the slightly different choice of which interpolation scheme is used for which camera and filter combination (Table 6) because of the different filters on Mastcam-Z. The onboard image-processing pipeline can run at the camera head’s full speed, writing the processed data stream directly into DEA memory, which contains 128 MB of Synchronous Dynamic Random-Access Memory (SDRAM) and 8 GB of non-volatile NAND flash memory per camera organized as a large image buffer, allowing images to be acquired without use of rover memory resources at the maximum camera data rate. The SDRAM is typically used as scratch space and to store file system information but can also be used as a small image buffer.

**Table 6:** Bayer pattern interpolation scheme used for lossy JPEG-compressed Mastcam-Z data.

Camera	Filter 0	Filter 1	Filter 2	Filter 3	Filter 4	Filter 5	Filter 6	Filter 7
Left	Malvar^a^	Red^b^	Red	Red	Red	Green^d^	Blue^e^	Identity
Right	Malvar	Red	Identity^c^	Identity	Identity	Identity	Identity	Identity

^a^Malvar means that interpolation using the algorithm of Malvar et al. (2004) is performed.

^b^Red means that bilinear interpolation of red Bayer pixels is performed; blue and green pixels are discarded.

^c^Identity means that no interpolation is performed; image is returned as a monochrome JPEG that was compressed from raw data with as-is Bayer values (because the Bayer filters are transparent at near-IR wavelengths; see Bell et al. 2017, Fig. 3).

^d^Green means that bilinear interpolation of green Bayer pixels is performed; red and blue pixels are discarded.

^e^Blue means that bilinear interpolation of blue Bayer pixels is performed; red and green pixels are discarded.

#### DEA Flight Software

DEA hardware functions are coordinated by the DEA instrument flight software (iFSW), which runs on the Microblaze. The DEA iFSW receives commands generated on Earth as dispatched through the rover, executes commands, and transmits any resulting data. The iFSW also implements onboard autofocus and “Z-stack” algorithms (for focus merges and range mapping; Edgett et al. 2012) and auto-exposure (Maki et al. 2003) for image acquisition, performs error correction on the contents of flash memory, and implements mechanism control and fault protection. The iFSW is essentially a copy of the DEA iFSW originally developed for the MSL Mastcam. The only software changes for the Mars 2020 implementation have been minor bug fixes, some enhancements to the autofocus algorithm, support for the specifics of the zoom and focus mechanisms, and the addition of a 2 × 2 pixel summing capability. The iFSW consists of about 10,000 lines of ANSI C code.

### Multispectral Filters

In addition to the Bayer RGB filters bonded onto the CCD and used to obtain “true color” (simulating human vision) by imaging through a broadband near-IR cut-off filter known as “Filter 0”, each Mastcam-Z filter wheel has 7 additional filters available for specific color applications (Table 3). The filters are interference filters made from Corning 7980 HPFS glass and were manufactured by Materion Precision Optics (formerly Barr Associates), the same manufacturer of the filters for MSL Mastcam. Band center wavelengths, band widths, peak in-band transmission, rejection band performance, and mechanical dimensions, flatness, irregularity, durability, scratch/dig specifications, and pinhole tolerances were all defined by the Mastcam-Z science and engineering teams. All filters have met their required specifications, as reported in detail in the companion Mastcam-Z pre-flight characterization and calibration paper (Hayes et al. 2021).

### Calibration Targets

**Fig. 8:**
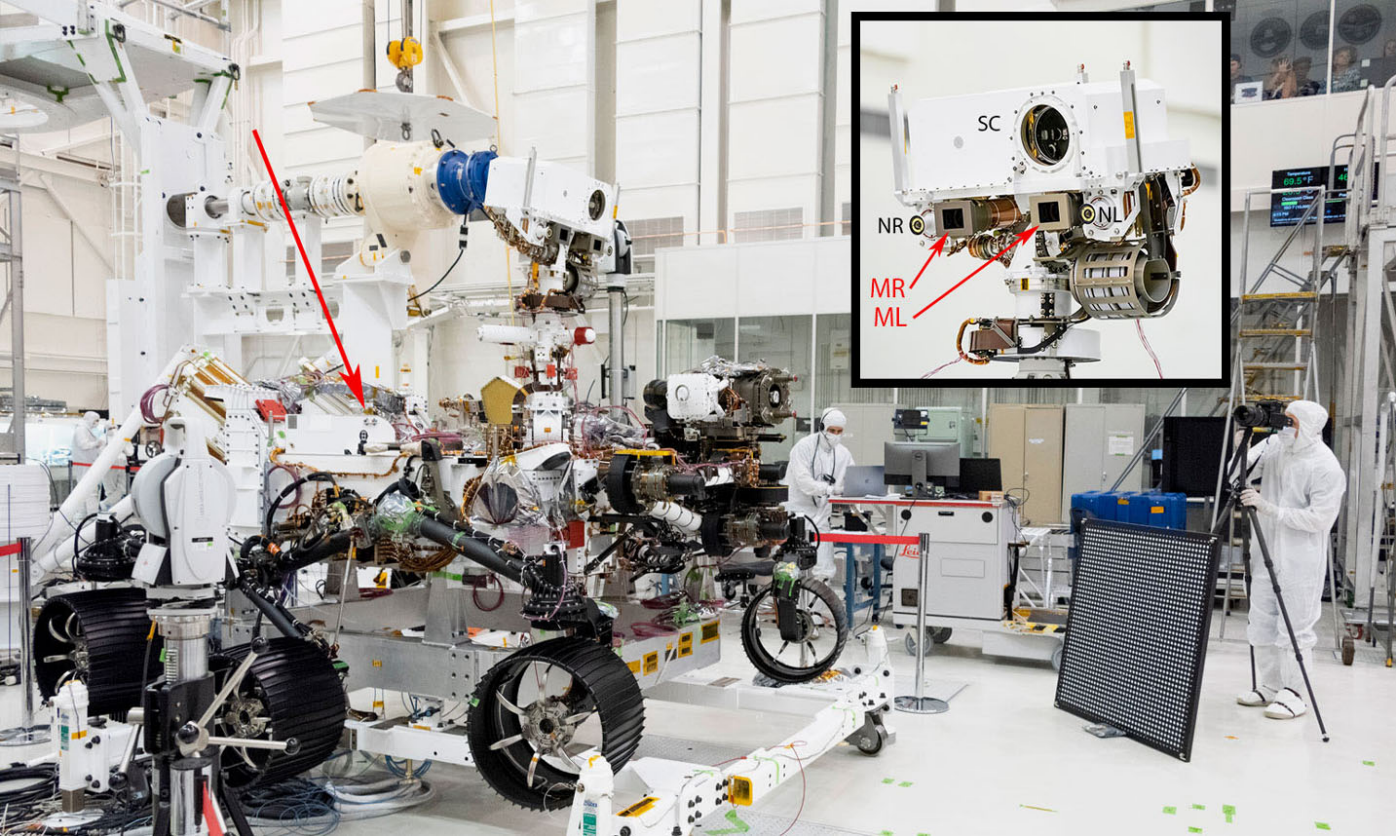
The Mars 2020 mission’s *Perseverance* rover, in testing at the Jet Propulsion Laboratory in July 2019. The inset shows the top of the rover’s Remote Sensing Mast assembly, with the Mastcam-Z left (ML) and right (MR) cameras as well as the rover’s two Navcams (NL, NR) and the mast unit of the SuperCam (SC) instrument, including the aperture for the Remote Micro-Imager (RMI) and other SuperCam systems (Maurice et al. 2020; Wiens et al. 2020). The red arrow shows the location of the Mastcam-Z Primary and Secondary calibration targets on the rover deck (see Fig. 4 and Kinch et al. 2020a)

Mastcam-Z uses two passive calibration targets, mounted on the RPFA box on the rover deck (Fig. 8), to enable tactical color calibration of the images. The targets provide a relative reflectance calibration technique similar to that employed on Mars Pathfinder (Reid et al. 1999; Bell et al. 2000), MER (Bell et al. 2003; 2006) and MSL (Bell et al. 2017), using similar kinds of passive targets with calibrated grayscale and color materials and a shadow-casting central gnomon. Imaging of the targets under similar viewing conditions (time, solar incidence angle) as images of surface scenes enables the rapid generation of tactically-useful relative reflectance calibration of the images for direct comparison with laboratory reflectance data without the need for a rigorous atmospheric correction. Based on lessons-learned from MER and MSL, a simple L-bracket Secondary Calibration Target (with a subset of identically-colored horizontal and vertical color patches) was added to *Perseverance* to allow better characterization of the rate and spectral influence of dust accumulation on the deck and targets. In addition, the Primary Calibration Target has strong embedded magnets under each color patch that repel airfall dust from the center of each patch (a heritage design used on Phoenix and MSL; see Leer et al. (2008) and Bell et al. (2017), respectively) to enable the properties of the most “clean” surfaces to be monitored. More details on the design, testing, fabrication, rover location and viewing angles, tactical use, expected level of calibration fidelity, and education/public outreach embellishments of the Mastcam-Z calibration targets can be found in Kinch et al. (2020a) and Kinch et al. (2020b).

### Integration and Articulation

The two Mastcam-Z camera heads are mounted onto a camera plate on the rover’s azimuth-elevation articulated Remote Sensing Mast (RSM; Fig. 8), which is a copy of the RSM used on the MSL *Curiosity* rover (Warner et al. 2016). The cameras are connected to the DEA by flex cabling that runs down the RSM and into the rover’s body (also known as the Warm Electronics Box, WEB). By convention, the “left” cameras are on the left side of the bar as seen by a viewer standing behind the “head” of the RSM (corresponding to -Y in the rover navigation or RNAV coordinate frame). The left and right Mastcam-Z instruments stereo baseline is 24.4 cm (Hayes et al. 2021), which will yield hyperstereo images for scientific and navigation purposes (for reference, the stereo separation or interpupillary distance between human eyes is ∼5 to 7 cm) and are inboard of the rover’s two Navigation Cameras (Maki et al. 2020). Because of the heritage RSM design, the cameras are not spaced symmetrically about the RSM azimuth rotation axis; the left Mastcam-Z is ∼0.5 cm farther from the axis than the right Mastcam-Z (Fig. 8). The Mastcam-Z camera heads are positioned relative to each other with a net toe-in angle of 2.3° (1.15° per camera), which, at 110 mm focal length, results in 100% image overlap at ∼6.1 m from the cameras and ∼63% overlap at infinity. The RSM provides the ability to point the cameras over ±181° in azimuth and −87° to +91° in elevation. When the RSM is deployed after landing and the camera plate is positioned in its nominal horizon-viewing elevation (0°), the Mastcam-Z cameras will be ∼2.12 m above the Martian surface.

While the Mastcam-Z camera optics and mechanisms are sealed and protected from contamination by dust and debris, there are no covers over the outermost optical surfaces (the leftmost fixed lens in Fig. 7) and light shade. During the cruise and entry, descent, and landing (EDL) phases of the mission, the ends of the light shades are stowed in a protective camera housing on the rover deck to help prevent contamination of the front lenses. After RSM deployment and during the mission, dust mitigation on the front lenses is achieved operationally by always pointing the cameras downward when not in use. Experience from MER and MSL shows that the front lenses and light shades will still get dusty and could accumulate material over time, but also that wind gusts will occasionally clean some or all of the dust off the lenses. Small drain holes have been added to the light shade to mitigate accumulation of dust or windborne particles in that volume. Expectation of variable dust contamination on the front optics is one of the motivators for periodic acquisition of new flat field calibration data using the Martian sky as a “flat” source (*e.g.*, Bell et al. 2017).

## Pre-Flight Verification, Validation, and Calibration

The Mastcam-Z flight model Camera Heads, DEA, and Calibration Targets each went through rigorous environmental and lifetime requirements verification and validation testing (V&V) at the “standalone” level prior to integration with the rover, and then went through a subset of V&V testing again together at the “system” level along with all of the other instruments and subsystems on the rover. In addition, the flight model camera heads went through a rigorous science calibration at the standalone level prior to integration with the rover (Hayes et al. 2021). V&V was performed at Malin Space Science Systems, Inc. (MSSS) for the Camera Heads and Electronics, and science calibration of the camera heads was also conducted at MSSS. V&V and spectroscopic/goniometric characterization of the Calibration Targets was conducted at the University of Copenhagen (Kinch et al. 2020a).

### Instrument-Level Testing

In addition to the flight model (FM) Mastcam-Z camera heads and DEA, two engineering qualification model (EQM) camera heads, two testbed unit (TBU) camera heads, and one TBU and one EQM DEA were built and delivered to JPL for development and qualification testing purposes. The TBU systems used non-flight grade electronics and other subsystems and were primarily for early software testing. The EQM camera heads consist of a flight-like opto-mechanical zoom and focus lens assembly, a flight-like filter wheel, and flight spare camera head electronics, all matching the flight design in form, fit, and function. The first EQM was used as a pathfinder for the FM camera head assembly and was subjected to functional testing, imaging performance testing at Earth-ambient and Mars-operational temperatures and pressures, pyroshock qualification testing, and mechanism life testing (to >2x expected mission life cycles). Both EQMs are being used for flight-like camera testing in the Mars 2020 Vehicle System Testbed at JPL.

The flight model camera heads and DEA also went through rigorous acceptance testing of their various subassemblies (electronics, optics/mechanisms, and software) prior to integration into the cameras and DEA, followed by the standard flow of random vibration testing, thermal vacuum testing, and instrument science calibration.

**Fig. 9:**
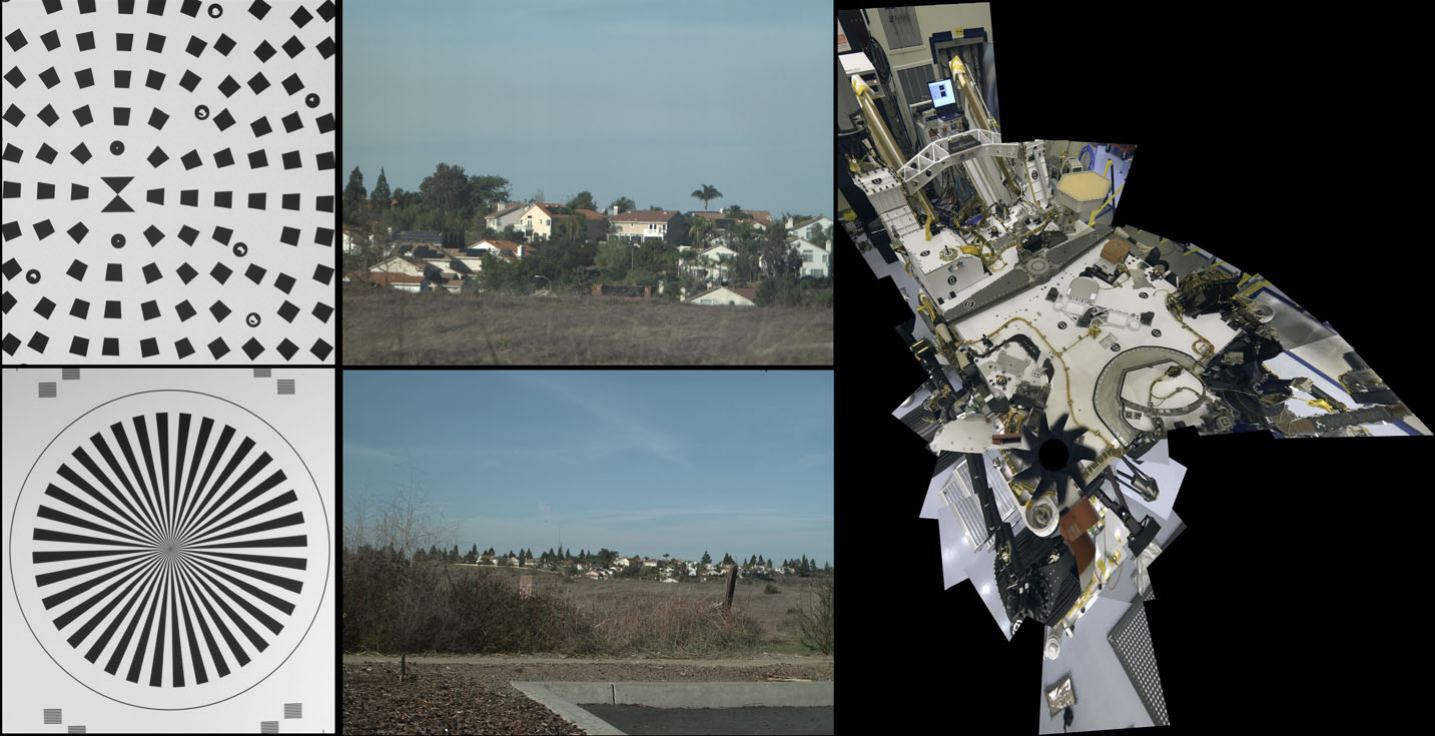
Examples of characterization and calibration images taken by the flight Mastcam-Z cameras during pre-launch testing in 2019 and 2020. (Upper left) Part of an MTF test target imaged at 100 mm focal length from a range of 7 m. The central “bow tie” feature is 5.6 cm tall; (Lower left) Part of a 57-cm diameter “Siemens star” MTF test target imaged at 34 mm focal length from a range of 5 m. (Upper and lower middle) Examples of distant targets imaged at 26 mm and 110 mm focal lengths. The houses on the top of the distant ridge are approximately 1 km away. (Right) Example 34 mm focal length 360° downward-pointing mosaic of the *Perseverance* rover’s deck, assembled from 53 individual 34 mm focal length images taken during ATLO testing at KSC in March 2020 for geometric mapping and RSM pointing verification

Science calibration of the Mastcam-Z camera heads measured, as a function of temperature and focal length where appropriate and possible: (1) absolute radiometric response; (2) system noise; (3) geometric distortion; (4) focus quality (MTF) over the field of view; (5) spectral response; (6) stray light susceptibility; (7) focus stepper motor count as a function of focus distance; and (8) zoom stepper motor count with image scale. Seven specific focal lengths were characterized in the most detail: 26, 34, 48, 63, 79, 100, and 110 mm. The calibration effort, the results of which are described in detail in the companion paper by Hayes et al. (2021), was a close collaboration among the Mastcam-Z science and engineering teams and relied on many of the procedures and software originally developed for MER/Pancam and MSL/Mastcam, fine-tuned to the specific requirements of the Mars 2020 Mastcam-Z investigation. For geometric and stereo data analyses, the team also utilized software developed for the European ExoMars 2022 Mission’s PanCam (Coates et al. 2017) and CLUPI (Josset et al. 2017) instruments. Examples of pre-launch characterization and calibration images taken with the Mastcam-Z flight cameras are shown in Fig. 9, and details on the results of Mastcam-Z pre-flight calibration tests are presented in Hayes et al. (2021).

### ATLO Testing

The flight model Mastcam-Z DEA was delivered to JPL in April 2019, followed by the camera heads and calibration targets in late May 2019. Upon receipt at JPL, the flight hardware underwent standalone vacuum bakeout, planetary protection bioassay, and functional testing prior to delivery to the Mars 2020 Assembly, Test, and Launch Operations (ATLO) team for integration with the rover.

During summer 2019, the Mastcam-Z hardware was mechanically and electrically integrated with the rover (Fig. 8). After integration, the cameras underwent additional functional testing using the full range of camera capabilities (focus and zoom lens motion, imaging targets at different focus and zoom positions and different RSM azimuths and elevations, video commanding, exercising of compression options, etc.). Particular to the Mastcam-Z investigation, ATLO tests included geometric calibrations that established Mastcam-Z pointing as a function of distance, focus, and zoom in the rover’s reference coordinate frame (including with reference to fiducial marks on the rover deck; Fig. 9) as well as determination (to the extent possible) of the position of other RSM-mounted instrument fields of view in Mastcam-Z images In fall 2019 the cameras also participated in rover system thermal vacuum testing to establish functional performance with relevant thermal boundary conditions, as well as rover surface Electromagnetic Interference/Electromagnetic Compatibility (EMI/EMC) tests to characterize the effects of potential sources of image data noise (Hayes et al. 2021). Just before delivery of the rover to the Kennedy Space Center (KSC) in Florida, EMI/EMC from/with other instruments (*e.g.*, helicopter, telecom subsystem, etc.) was characterized and found to be undetectable or acceptably low. Additional “baseline tests” (acquiring dark frame images) were conducted throughout the ATLO campaign, including before and after transport of the rover to KSC for launch preparations in February 2020, and with the flight Radioisotope Thermoelectric Generator (RTG) installed; all of these tests showed nominal performance of the instruments. Final inspection and cleaning of the front optics was performed at KSC in late March 2020 prior to the final stowing of the RSM for launch, which occurred successfully on July 30, 2020.

## Mission Operations and Data Products

### Operational Considerations

While images at any focal length between 26 mm and 110 mm can be commanded, the expectation during Mars surface operations is that images will nominally be acquired at one of the seven well-calibrated zoom settings noted in Sect. 4.1. Additionally, for nominal stereo or full-filter multispectral imaging, both cameras will be set to the same zoom position. In situations where images are to be acquired with only one camera, neither is intrinsically preferable. Rather, other considerations would dictate the choice of which camera to use (*e.g.*, desire for a specific multispectral or solar filter; desire for the closer alignment of the left camera to the SuperCam RMI field of view, etc.)

Mastcam-Z uplink, downlink, and archiving operations will be led from the Mastcam-Z Science Operations Center (SOC) at Arizona State University (ASU) in Tempe, Arizona. Downlink operations and data archive development work will be performed at ASU. Uplink operations will be performed at MSSS in San Diego, California. Dedicated operations staff at ASU and MSSS will fill the primary uplink, downlink, and archiving roles associated with both tactical and strategic operational planning, instrument health assessment, and data analysis, but their work will be augmented by the significant involvement of trained science co-investigators and collaborators on the team, including many undergraduate and graduate students and postdocs at ASU and other team institutions.

The Mars 2020 Project is considering co-locating instrument operations, science team members, and rover engineering teams at JPL in Pasadena during the first 3-4 months after landing on February 18, 2021. However, the 2020 CoVID-19 pandemic could modify those co-location plans. Regardless of their physical locations on Earth, many of these personnel will likely be living on “Mars time” (on the 24 hour, 39 minute Martian diurnal cycle) to maximize operational efficiency while conducting the initial assessment of the rover and the landing site. After that initial period, the entire operations team will transition to conducting operations remotely from their home institutions. Mastcam-Z operational facilities are already developed at ASU and MSSS and have been used for Mars 2020 initial operations tests. Many of these facilities were recently used for MER/Pancam operations and are currently being used for MSL MAHLI, Mastcam, and MARDI rover operations, as well as operations for other missions.

Mastcam-Z is expected to participate in all five types of “sol templates” currently envisioned by the Mars 2020 Project’s operations team (Stack et al. 2020): (1) traverse and approach, (2) site reconnaissance (remote sensing science), (3) arm manipulation and contact science, (4) coring/caching and contact science, and (5) recharge/telecom. Mastcam-Z science team members and their collaborators will support the overall Mars 2020 investigation through participation in sol-to-sol tactical operations planning and downlink assessment, and/or strategic planning for drive paths and regions of interest in which to focus future potential *in situ* sampling and coring/caching.

### Commanding, Sequencing, and Downlink Assessment

The Mastcam-Z operations staff will perform the critical engineering tasks involved with Mastcam-Z uplink sequencing/commanding and downlink data processing and reporting. This work includes the actual writing of command sequences, checking for flight rule violations, monitoring instrument health and performance, and processing data to be passed along to the science and engineering teams. Additional staff tasks include: strategic operations planning for Mastcam-Z usage on subsequent sols; potentially testing new sequences/techniques in the rover testbed at JPL; and validation and archiving of Mastcam-Z data for the NASA Planetary Data System (PDS). The Mastcam-Z Ground Data System (GDS) hardware and software leverage developments and refinements of the GDS from operating the MER Pancam and MSL Mastcam cameras, as well as many of the software tools developed for MER Pancam and MSL Mastcam, MAHLI, and MARDI at ASU and MSSS. Software tools developed or refined for Mastcam-Z will enable rapid image calibration as well as creation of other derived products (e.g., false-color images made from the near-UV and/or near-IR filters in Table 3), mosaics, and stereo anaglyphs) all on a tactical timeline (i.e., within minutes to a few hours of the receipt of each downlink) to help inform decisions by the science and engineering teams. Other software tools developed or updated for Mastcam-Z enable stereo data visualization, and streamline data validation for public release and archival purposes.

### In-Flight Calibration Plans

The Mastcam-Z cameras have undergone simple instrument check-outs (bias and dark current assessment) that have verified instrument health health during the ∼7-month cruise to Mars. Once on the Martian surface, functional tests to verify camera performance and initial Mastcam-Z scene imaging will occur as early as possible during the Surface Operations Transition (known as “SOX”) phase of the mission. Early images will include verification of the pre-flight bias and dark current, multispectral characterization of the calibration targets (prior to significant dust accumulation), and characterization of the geometric, focus, and zoom performance of the cameras as a function of object distance and camera temperature, including imaging of geometric fiducial targets on the rover deck. Occasional calibration images, such as flatfields using the Martian sky as a target, stray light characterization using images of the sky near the Sun, and dark current/hot pixel monitoring sequences, will be conducted on a semi-regular cadence, based on previous MSL and MER experience (*e.g.*, Bell et al. 2006a, 2006b, 2017). Calibration target images will typically be acquired close in time with every narrowband multispectral imaging sequence that they are designed to help calibrate.

### Data Processing and Products

#### Raw Data Products and Formats

Mastcam-Z science data consist of full-frame and sub-framed images, thumbnail images (about 1/64th the number of pixels of a full frame), Z-stack products, or compressed video Groups of Pictures (GOPs) stored in DEA flash memory. There is enough DEA space to store up to ∼4,000 uncompressed full-frame Mastcam-Z images for each camera. Raw data are expected to be transferred to the rover and downlinked in four primary formats: (1) Color JPEG images and thumbnails: Bayer pattern interpolated, 8-bit companded files in standard JPEG format (band-separated, YCbCr components in 8×8 coefficient blocks); (2) Losslessly-compressed images: predictive losslessly-compressed, band-sequential 8-bit companded raster images, without Bayer pattern interpolation; (3) Compressed color videos: Bayer pattern interpolated, 8-bit companded, lossy JPEG-compressed images concatenated into 16-frame motion-JPEG GOPs (termed a “video”); (4) Raw 11-bit images: 11-bit raster files (stored in 16-bit format) with no compression or interpolation applied. Bell et al. (2017) provide additional details on these data types, which are the same as the data product types created by the Mastcam, MAHLI, and MARDI cameras on MSL. All images will include instrument and spacecraft telemetry headers.

The baseline operating procedure for Mastcam-Z, based on MSL Mastcam experience, will be to acquire and store each image uncompressed in the DEA, giving the science team many options for the eventual downlink of those images. If expected downlink data volumes are low and an image is needed for tactical planning, a medium quality JPEG can be returned; later, when downlink data volumes are higher, a higher-quality JPEG or a losslessly-compressed version of the same image can be returned. We expect most data downlinked to be compressed JPEG images with Quality Factors from 65 to 95 (based on MSL/Mastcam experience) and thumbnails. The Mastcam-Z operations team will manage the contents of the DEA’s onboard storage volume, act on requests from the Mars 2020 Science Team to downlink stored data (or re-downlink at higher fidelity), and alert the science and engineering teams when data are going to be deleted.

#### Onboard Data Processing

The Mastcam-Z instrument flight software is based on the same software used by the MSL Mastcams. In addition to the capabilities listed in Sect. 3.3.2, we will also use the existing MSL Mastcam software to control RSM pointing and gather rover/spacecraft metadata, and the Mars 2020 mission’s GDS to catalog and downlink images to Earth.

#### Data Quantity

The quality and quantity of images to be downlinked from Mastcam-Z are highly dependent on the details of the mission’s field site, the cadence of mission activities, and its downlink bandwidth capabilities. The latter is being designed to be largely dependent upon data relay through orbiting assets such as the Mars Reconnaissance Orbiter (MRO), MAVEN, and Trace Gas Orbiter (TGO). A typical “day in the life” of Mastcam-Z could include drive-direction color mosaics, images of the Sun for atmospheric opacity, remote sensing (*e.g.*, multispectral imaging through narrowband filters) of science targets seen in Navcam images, images of the Mastcam-Z calibration targets, and perhaps a 180° to 360° color panorama. Mastcam-Z will also provide context imaging for other payload elements and vehicle assessment imaging for rover engineers. In case of off-nominal downlink capabilities (such as the potential ∼15 Mbits/sol for downlink communications scenarios that involve only the use of the rover’s High Gain Antenna), the outstanding performance of the MSL heritage JPEG compressor at lower quality factors (*e.g.*, Malin et al. 2017; Bell et al. 2017), along with the use of color thumbnail images, should still enable sufficient imaging to conduct tactical rover science operations.

Because of high-level investigation goal similarities, one way to estimate how many images Mastcam-Z will acquire and return to Earth is by analogy to MSL Mastcam downlink, scaled to the 669 sol Mars 2020 Prime Mission plus 20% margin to account for the enhanced stereo capabilities of Mastcam-Z that are expected be used to support rover operations. Based on PDS-archived data from MSL, this suggests that, over the course of the Mars 2020 Prime Mission, Mastcam-Z could acquire ∼32,000 thumbnails, ∼25,000 RGB full-frame images and ∼6800 narrowband multispectral images, for a total estimated prime mission raw data volume of ∼120 Gbits (∼175 Mbit/sol).

#### Rapid Data Products for the Mars 2020 Team, the Public, and the Scientific Community

**Fig. 10:**
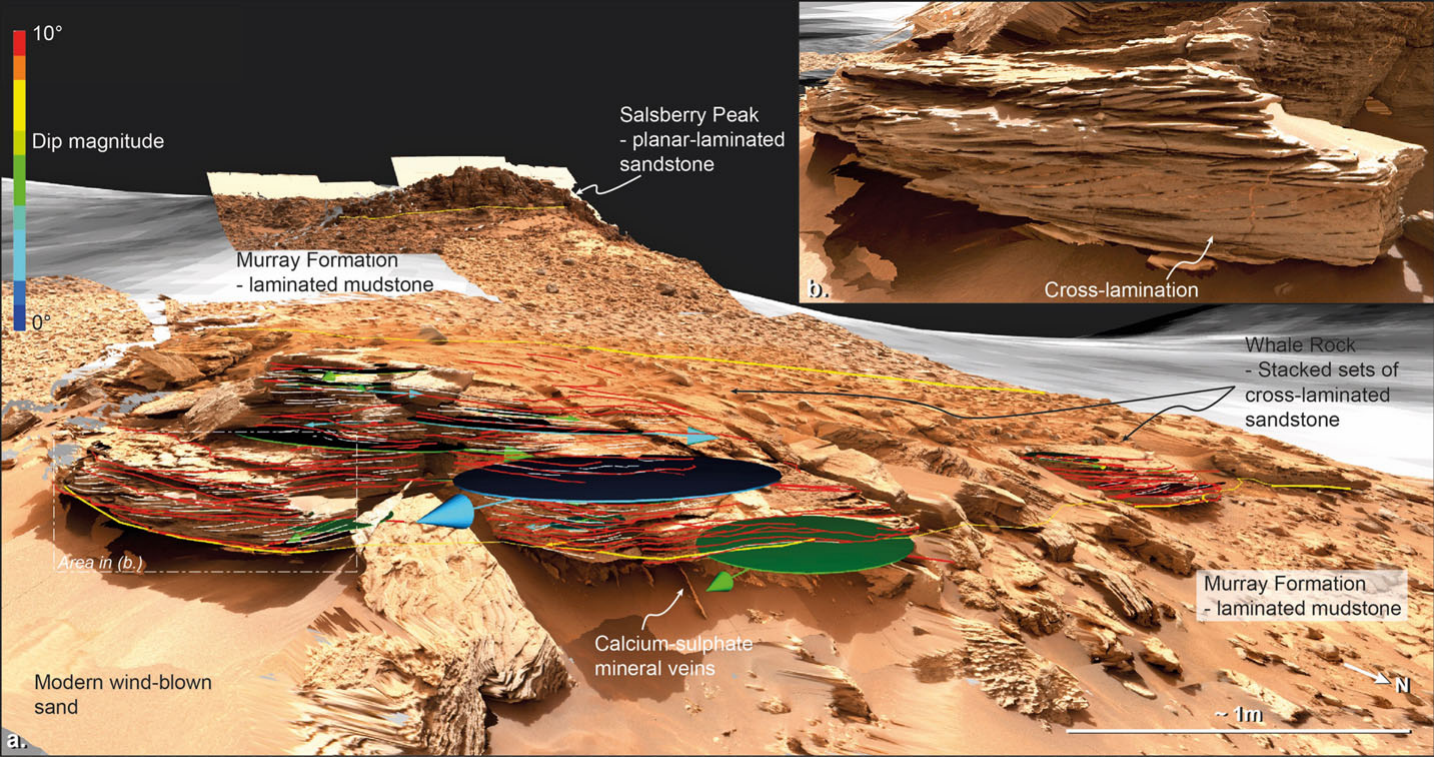
Example of reconstructed and geologically interpreted 3-D stereo measurements using MSL *Curiosity* rover Mastcam images of the Whale Rock target at the Pahrump Hills outcrop in Gale crater. Stereo data sets like this enable the assessment of quantitative sedimentological parameters like strikes, dips, and bedding plane orientation and thicknesses that can directly inform specific past geologic origins and environments. The reconstruction here was generated using the same data visualization and analysis tools to be used for geologic interpretations of Mastcam-Z stereo image products (“PRo3D”; Barnes et al. 2018). Mastcam-Z can obtain stereo data at up to 4 times higher spatial resolution than MSL/Mastcam.

The Mastcam-Z team is committed to the rapid release of high-fidelity color images and mosaics to the Mars 2020 science team as well as to the general public. Planned derived data products for use by the science and engineering teams include: (a) RGB and multispectral color panoramas and mosaics; (b) red/blue anaglyphs, stereo mosaics, stereo pairs, DTMs, terrain meshes, and contour maps; (c) measurements and analyses of reflectance ‘spectra’ from multispectral observations of surface targets; (d) quick-look multispectral parameter images/mosaics of potential Fe-bearing and hydrated mineral detections; (e) annotated mosaics indicating textural, structural, and stratigraphic relations and interpretations of outcrops, rocks, and fines; (f) 3D geologic annotations and measurements to quantitatively characterize the 3D geometry of sedimentary rock outcrops and sedimentary structures (*e.g.*, Fig. 10; Barnes et al. 2018; Banham et al. 2018); (g) measurements and analyses of atmospheric opacity, dust properties, airfall dust deposition levels on calibration targets and other rover deck instruments (*e.g.*, MOXIE, MEDA), and meteorological and astronomical phenomena; and (h) time-lapse and motion videos of the *Perseverance* rover, its *Ingenuity* technology-demonstration helicopter, and their immediate environs. If downlink data volume constraints allow, it should also be possible to achieve higher than nominal spatial resolution on Mastcam-Z targets of interest using dithering or other relevant “super resolution” techniques (*e.g.*, Bell et al. 2006b).

Mastcam-Z raw images (as JPEGs) will also automatically be made available to the public using the Mars 2020 Project’s web site, within minutes of their arrival on Earth. In addition, the Mastcam-Z team will supplement these raw publicly released JPEGs with other derived data products (*e.g.*, false color images, mosaics, stereo anaglyphs) posted onto a publicly accessible Mastcam-Z web site at ASU (http://mastcam-z.asu.edu). The Mars 2020 mission’s main web sites at JPL and NASA will also host captioned Mastcam-Z images and press releases, as appropriate.

The archival science products of the Mastcam-Z investigation will be time-ordered individual images, compliant with the most recent PDS standards. The Mastcam-Z team will archive validated raw and derived data products as soon as feasible, but no later than 6 months after a given data product is received on Earth. In addition, all other images acquired by the flight cameras (from preflight calibration and testing, ATLO, and cruise), along with appropriate documentation, will be archived in the PDS.

## Summary

The NASA *Perseverance* rover’s Mastcam-Z variable focal length, multispectral, and stereoscopic imaging investigation is designed to help achieve a variety of Mars 2020 mission goals related to the geology, composition, current environment, and past habitability of the rover’s field site in Jezero crater. The Mastcam-Z investigation’s three main goals are: (1) to characterize the overall landscape geomorphology, processes, and the nature of the geologic record (mineralogy, texture, structure, and stratigraphy) at the rover field site, especially as they pertain to past or present habitability; (2) to assess current atmospheric and astronomical conditions, events, and surface-atmosphere interactions and processes; and (3) to provide operational support and scientific context for rover navigation, contact science, sample selection, extraction, and caching, as well as high-resolution imaging and video support for the other selected Mars 2020 investigations. We have defined 12 specific detailed investigation objectives associated with these goals (Table 2). These 12 objectives motivated specific observations to be obtained and instrument functional requirements that guided the design, testing, and calibration of the flight hardware.

Mastcam-Z hardware consists of dual camera heads separated by 24.4 cm and toed-in by 1.15° each, mounted on the rover’s fully-actuatable Remote Sensing Mast ∼2.12 m above a flat horizontal plane defined by the bottom of the rover’s wheels; a Digital Electronics Assembly mounted inside the rover body that provides the camera heads with power, commanding, data processing, data storage, and an interface to the rover computer; and two passive grayscale/color calibration targets mounted on the rover’s deck.

The camera heads are an essentially identical pair of focusable, 4:1 zoomable 1648×1214 pixel CCD imagers that provide broadband red/green/blue (RGB), and narrowband 400-1000 nm data in 11 unique narrow bands as well as color solar imaging capabilities. The cameras provide continuously variable fields of view ranging from 25.6° × 19.2° (26 mm focal length at 280 μrad/pixel) to 6.2° × 4.6° (110 mm focal length at 66.7 μrad/pixel) and are thus capable of resolving (≥5 pixels across) features larger than ∼0.7 mm in size at 2 m and ∼3.3 cm in size at 100 meters.

This contribution has provided details on the goals, objectives, requirements, designs, fabrication, testing, integration, and expected operations and data products for the Mastcam-Z instrument. Companion papers by Kinch et al. (2020a) and Hayes et al. (2021) provide details regarding the Mastcam-Z calibration targets and pre-flight characterization and calibration.

## Data Availability

Data and material described here are either already in the public domain, are being published via Open Access, or are being archived in the NASA Planetary Data System for general community and public access.
